# Force-Accelerated Autocatalysis in a Knoevenagel Condensation Reaction during Ball Milling

**DOI:** 10.1021/jacs.5c23405

**Published:** 2026-07-10

**Authors:** Kathleen R. Floyd, Emmanuel C. Nwoye, Lizette Mella, Paulina V. Gonzalez, Jonathan R. Felts, James D. Batteas

**Affiliations:** † Department of Chemistry, 14736 Texas A&M University, College Station, Texas 77843-3255, United States; ‡ Department of Mechanical Engineering, Texas A&M University, College Station, Texas 77843-3123, United States; § Department of Materials Science and Engineering, Texas A&M University, College Station, Texas 77843-3127, United States

## Abstract

The kinetics of a Knoevenagel condensation between vanillin and barbituric acid has recently provided insight into the differences between mechanical and traditional solution-based synthetic methods. The solution-based reaction follows first-order reaction kinetics, while mechanochemical reaction kinetics follow a sigmoid pattern, with a rapid acceleration of reactivity following a slow induction period. Previous works theorize the source of the reaction acceleration to be either particle fracture evolution or changes to the reactant’s rheological properties. Here, we examined the reaction kinetics in stainless steel, Teflon, zirconia, and aluminum reaction vessels using different milling frequencies to determine the role of reagent mechanics under varying mechanical environments. Reaction vessels with interchangeable midsections and end-caps of different materials were used to discern the role of jar/ball material surface energy and localized shear vs normal loading forces on the reaction kinetics. The kinetics remained sigmoidal regardless of milling jar/ball materials, milling frequency, and observed rheological changes. Based on a kinetic energy model, the reaction is consistent with a force-accelerated autocatalytic process. High mixing in a low-force environment (Teflon) resulted in conversion ∼8x higher than high-force environments (stainless steel), which also showed more ready formation of solid volumes of product that were not free-flowing, reducing yields, suggesting that optimizing interfacial adhesion, impact force, and the relative amounts of shear vs normal forces yields significant rate improvements. Previously reported “cohesive states” likely arise from conditions of shear flow in reaction systems, making the methods introduced here broadly applicable to a host of mechanochemically accelerated chemical systems.

## Graphical abstract

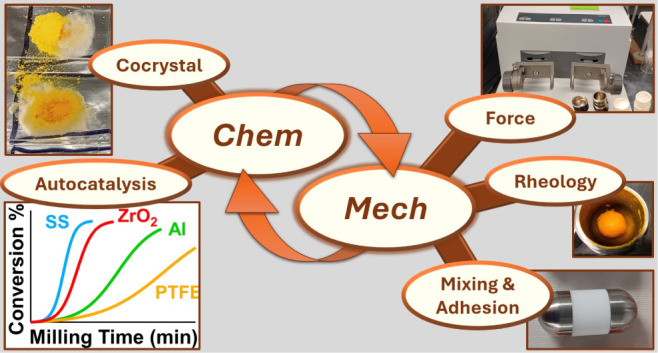

## 1 Introduction

Traditional methods of chemical synthesis typically rely on the dissolution of reagents in a solvent, reflux heating, and purification of the desired product from the mixture.^1^ Intense study over the past century has refined these standard techniques to a high degree, enabling chemists to control and predict reaction outcomes for a broad range of chemical manufacturing processes on industrial scales.^1^ While mature, the traditional thermochemical synthetic approaches face new challenges in the modern era of increased efficiency and sustainability. For example, 85% of chemicals currently employed for industrial manufacturing are considered hazardous and environmentally damaging.^2^ Recycling recovery rates are minimal, with anywhere from 20% to 50% of solvent lost to the environment.^2^ This has prompted regulatory action from governmental authorities, as evidenced by the recent ban on methylene chloride in 2024.^3^ Moreover, dissolving the reagents in a solvent and then employing energy-intensive methods to remove the solvent later results in significant energy waste and inherent inefficiency.^4^ This process also limits the reaction scope if a suitable solvent matching reaction precursor solubility cannot be identified.^5^

Ongoing efforts are being aimed at addressing these issues.^6^ One innovative approach removes solvents and traditional reaction vessels to instead focus on achieving reactivity by mixing chemical feedstocks in the solid state with substoichiometric to no solvent use. This approach is known as mechanochemistry, wherein mechanical force and the associated energetic environment generated under force (usually applied by grinding and crushing reagent precursors together) are utilized to alter reaction energy landscapes and reduce activation barriers, affording unique and energetically efficient syntheses. Mechanochemistry was recently identified by the International Union of Pure and Applied Chemistry (IUPAC) as one of the top 10 chemical innovations expected to change the world, with many elements of mechanochemistry aligned with the 12 principles of green chemistry.^7,8^ Particularly in the area of organic chemistry, mechanochemical approaches have been increasingly applied to enhance reaction performance in terms of yield, sustainability, substrate scope, and overall efficiency, enabling a broad range of chemical transformations.^9–14^

Despite its power as a synthetic method, quantitative predictions of reaction rates in mechanochemistry lag behind solvothermal methods, as it requires developing a clear understanding of the entangled roles of reagent powder mechanics in the solid state, reactor, and reaction environment, the interfacial interactions between the reagents and the reactor surfaces, and how mechanical forces are applied to, and energy dissipated into, the reagents. These factors can make reactivity difficult to optimize and predict due to outcomes that can vary from those seen when traditional processes are used, alternatively enabling access to novel products or complicating attempts to translate traditional reactions to mechanochemical processes.^5,15–17^

Here, we examined a Knoevenagel condensation reaction between vanillin and barbituric acid as a testbed system to explore the roles of these factors in more detail (see Scheme 1). The Knoevenagel condensation is a significant organic transformation for C=C bond formation, typically performed between aldehydes and ketones to yield α,β-unsaturated ketones.^18,19^ This classic reaction is important for the synthesis of a variety of high-value dyes^20–22^ and fluorophores,^23^ while serving as an important intermediate step in the formation of natural products,^24,25^ pharmaceutical compounds,^26^ polymers,^27^ insecticides,^25^ and pesticides.^25^ Furthermore, the vanillin and barbituric acid system has served as an important mechanochemical model for examining the energetics of milling processes,^28,29^ assessing scalability using twin-screw extrusion,^30^ studying kinetics both ex-situ and in situ,^28,31,32^ and demonstrating reactivity driven by cocrystal formation under both neat and liquid-assisted grinding (LAG) conditions.^31^

**Scheme 1:**
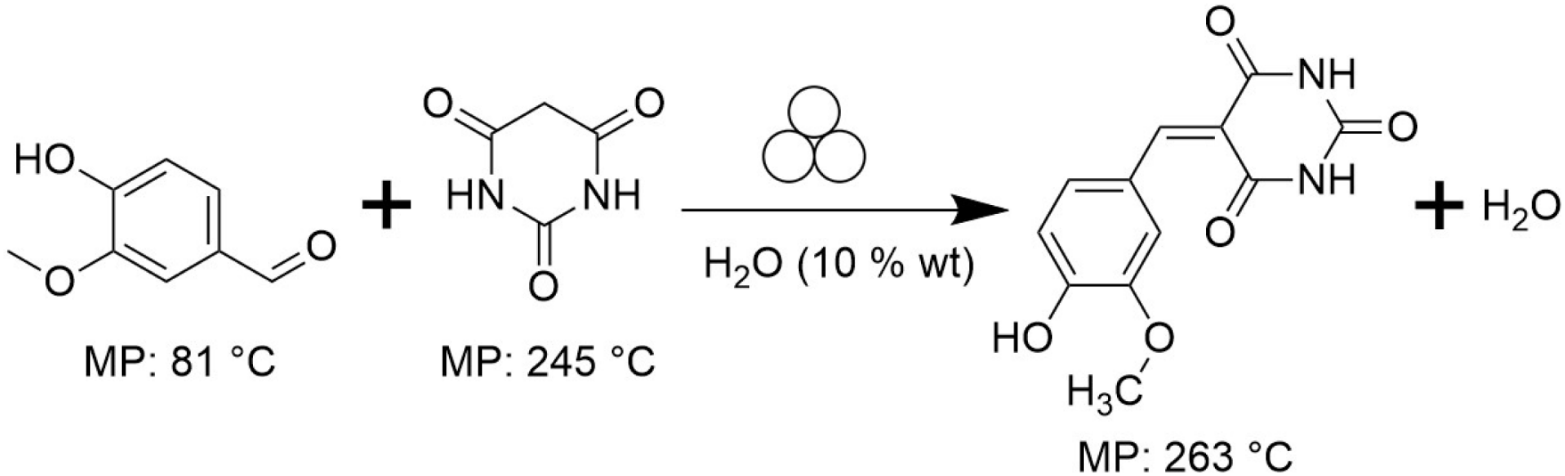
The Mechanochemical Reaction Between Vanillin and Barbituric Acid.

As shown previously, the reaction in aqueous solution takes approximately 28 days to reach completion, while by milling reagents directly in a mixer mill, this reaction can reach completion within 40 min, with substoichiometric amounts of water serving as the catalyst.^32^ Interestingly, the kinetics of the mechanochemical transformation exhibit a sigmoidal relationship, with slow product formation occurring during an induction period, followed by a period of rapid product formation in a “positive feedback” process lasting 3–7 min, after which near-quantitative conversion is obtained (Figure 1).^28,32,33^ This differs from the kinetics of the solution reaction, which follows a simple first-order process.^32^

**Figure 1:**
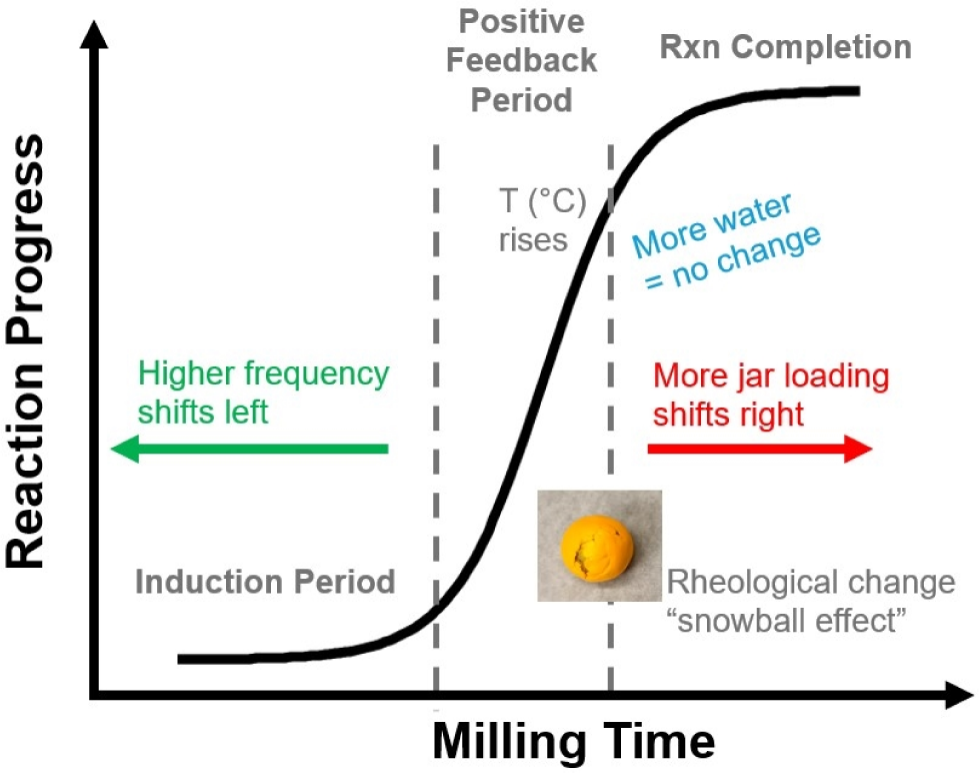
Diagram summarizing current knowledge about the kinetics of the condensation reaction between vanillin and barbituric acid in a Retsch MM 400 mixer mill.

Previous studies by Hutchings et al. showed that this positive feedback period depended on milling frequency,^32^ where a positive feedback period was not observed at 15 Hz (with correspondingly slow conversion, reaching only 20% after an hour), yet was observed at 25 Hz after ∼30 min, as well as at 30 Hz after ∼13 min of milling.^32^ For both 25 and 30 Hz, the positive feedback period started at ∼20% conversion and ended at 100% conversion soon thereafter.^32^ Moreover, it was also observed that the positive feedback period began earlier in the milling process at lower reagent loadings, and it did not change with increasing the amount of initial water, or with pregrinding of the reagent materials, which would have suggested that particle breakdown was responsible for the sudden onset of reaction. These findings set this reaction apart from recent literature, where premilling of reagents has been shown to be key to controlling reaction rates and the onset of the feedback period.^17^ Lastly, it was not found to be an effect that could be entirely explained by changes in measured jar temperature, although this was observed to rise during the feedback period and likely still contributes to the kinetics.^32^ These observations are summarized in Figure 1.

To account for these observations, the positive feedback period was suggested to correlate with a “cohesive state” wherein the reagents cake around a milling ball in a tight “snowball” formation, enabling higher temperatures around the ball and generally enhanced reactivity.^32^ Carta et al. modeled this behavior phenomenologically, suggesting that the induction period is due to the need to first form the reagent layer around the milling ball.^33^ This cohesion process continues during the positive feedback period, wherein far more reagents experience collisions with sufficient energy to overcome the activation barrier in the condensed state than they can without this state as a free-flowing powder, thus leading to the rapid reaction.^33^

Given that prior work has suggested that the rheology of the reaction system has a significant impact on the reaction kinetics, we set out to systematically connect the kinetics to the impact forces experienced by the powder (modeled by applying classical mechanics and using known frequency, ball size, and material mechanical properties), surface adhesion, mixing, effectiveness, and force directionality. This was achieved by controlling the milling material and the material location within the vessel.

As will be shown, the reaction does not consistently exhibit the formation of the “snowball” in stainless steel. Moreover, a “snowball” is not observed in milling jars of other materials (e.g., PTFE or Al), with only a single occurrence in zirconia, although a similar positive feedback period occurs in all systems. Mack and coworkers also saw conversion for this same reaction, without the formation of a cohesive state, but did not delve into the details of the mechanics.^34^ More recent reports suggest the reaction may proceed through a cocrystal intermediate and eutectic formation.^31^ We build on this foundation and model the kinetics as a force-accelerated autocatalysis of new product by existing product rather than having a direct dependence on the material rheology.^32,33^ This model suitably fits the data throughout the various materials and frequencies studied and excludes the snowball effect as the dominant cause of rate increase during the feedback period, and instead suggests a more overarching explanation of shear flow of the reagents (facilitated by interfacial adhesion to milling surfaces) as the key element in forming this precursor cocrystal. As such, we exploit the unique adherence of reagents to the different milling reactor materials to probe the effects of varying milling jar regions on reaction progression and mixing, revealing that shear forces along the sides of the jar are more effective at driving the reaction when the ball movement is confined. Given that a host of other important chemical systems reported in the literature invoke a “cohesive state” ^35–37^ or autocatalytic precursor state^38^ (including cocrystals)^39^ as a central element, our studies demonstrate new methodologies for modeling the role of force on gram-scale chemical reactions in vibratory mills, analyzing the interplay between force and mixing, and measuring the effect of different types of forces on reaction outcomes.

## 2 Materials and Methods

Chemicals were purchased from BeanTown Chemical and used without further purification. Vanillin was stored under nitrogen gas after each use to help prevent degradation (see Section 1 of the Supporting Information for further details).

### 2.1 Nuclear Magnetic Resonance Spectroscopy (NMR)

^1^HNMR spectra were obtained with either (1) a Bruker Avance Neo 400 instrument equipped with a 400 MHz Ascend magnet, an automated tuning 5 mm broadband iProbe, and a 60-position SampleXpress sample changer or (2) a 500 MHz Varian system equipped using a Varian VnmrS console equipped with an Oxford magnet and 5 mm 1H [X] broadband and [^1^H/^19^F] [X] switchable probes. Reference data for the product are available in Section 2 of the Supporting Information.

### 2.2 Powder X-ray Diffraction (PXRD)

The sample was placed in the sample holder of a two-circle goniometer, enclosed in a radiation safety enclosure. The X-ray source was a 1 kW Cu X-ray tube, maintained at an operating current of 40 kV and 25 mA. The X-ray optics were configured in the standard Bragg–Brentano para-focusing mode with the X-ray diverging from a DS slit (1 mm) at the tube to strike the sample and then converging at a position-sensitive X-ray Detector (Lynx-Eye, Bruker-AXS). The two-circle 218 mm diameter θ-θ goniometer was computer-controlled with independent stepper motors and optical encoders for the θ circle, with the smallest angular step size of 0.0001 to 2θ. The software suite for data collection and evaluation was Windows-based. Data collection was automated by the COMMANDER program by employing a DQL file. Data were analyzed by the program EVA. Further information is available in Section 2 of the Supporting Information.

### 2.3 Milling Jar Specifications

Trials employed stainless steel (SS) milling jars (25 mL) and associated stainless steel grinding balls (15 mm, 13.40 g) purchased from Retsch, along with grinding balls (12.7 mm, 8.55 g) made of corrosion-resistant 316 stainless steel purchased from McMaster-Carr. Zirconia (ZR) jars (25 mL) were made by Retsch, and zirconia grinding balls (12.7 mm, ∼6.0 g) were purchased from McMaster-Carr. PTFE milling jars (25 mL) and aluminum (AL) milling jars (25 mL) were manufactured in-house to match the internal dimensions of the Retsch models using chemical-resistant PTFE and multipurpose 6061 aluminum purchased from McMaster-Carr; associated milling balls (12.7 mm, 2.99 g (AL) & 2.30 g (PTFE)) of identical material were purchased from McMaster-Carr. Inserts for commercial 25 mL vessels made of SS and PTFE with 10 mL internal volumes, isolating each material to different regions of the vessel, were manufactured in-house using 316 stainless steel and chemical-resistant PTFE purchased from McMaster-Carr. More information on jar design and use is available in Section 2 of the Supporting Information. The insert designs are part of a provisional patent.^40^ The temperature of the milling jars and balls was measured by two approaches. Using an IR thermal camera (FLIR TG165-X) and using a K-type thermocouple (−40–1000 °C) attached to a digital multimeter (Extech Instruments EX355). Temperatures of the jars during milling generally range from 30–35 °C with the SS milling balls reaching maximum temperatures of 35–42 °C (see Supporting Information Section 10).

### 2.4 General Procedures for Reaction Kinetics Measurements

#### 2.4.1 Synthesis

Synthesis of 5-(4-hydroxy-3-methoxybenzylidene)­pyrimidine-2,4,6­(1H,3H,5H)-trione was performed in a Retsch MM400 mixer mill operating at a frequency of 30 or 25 Hz using the materials described in Section 2.3.

In a typical experiment, the milling jars and balls were first cleaned with acetone, water, and Alconox soap, followed by a rinse with DI water and drying at room temperature. Then, the milling ball (see Section 2.3), vanillin (1.9 mmol), barbituric acid (1.9 mmol), and water (53 μL, 10% wt) were loaded into a 25 mL milling jar (see Section 2.3). Jars were then placed on a Retsch MM400 mill, and milling was performed for the desired run time at either 25 or 30 Hz. Upon reaction completion, the mixture in the jars was photographed, and a small amount of crude product (typically 5–25 mg) was collected from multiple jar regions and dissolved in DMSO-*d*_6_ (750–800 μL) between 3 and 7 min following the end of the run. Conversion was measured by ^1^H nuclear magnetic resonance spectroscopy (NMR) (see Section 2.1). This process was repeated until all run times had been measured. While more experimentally costly in terms of time and resources, this methodology ensures that the results are unaffected by cooling or other potential factors that could interfere with the kinetics during milling pauses resulting from periodic sampling.^41^ Control experiments revealed that this precaution was well taken, as the reaction could continue slowly in the solid state upon pausing the milling process, thus the crude product was dissolved in *d*_6_-DMSO within 3–7 min upon removal from the jar and taken for analysis (see Section 3 of the Supporting Information). The reaction proceeds slowly in solution, with conversion changes of less than 5% observed after a day in solution. Thus, NMR measurements obtained within 12 h of dissolution are within the methodological error.

#### 2.4.2 Defining the Onset of the Positive Feedback Period

Former studies define the beginning of the positive feedback period by visualizing the original % conversion vs time graph. To be more quantitative, we have chosen to define this time using the second derivative of the derived kinetic fit of the data described in Section 3.4 and the % conversion reached by the % conversion fit value at that time point. Further details on this process can be found in Section 4 of the Supporting Information.

### 2.5 General Procedures for Reactions Confining Materials to Specific Milling Jar Regions

In a typical experiment, in-house designed milling jar inserts (described in Section 2.3 and Section 2 of the Supporting Information) were cleaned with acetone, water, and Alconox soap, followed by a rinse with DI water and drying at room temperature. Then, they were loaded with vanillin (0.76 mmol), barbituric acid (0.76 mmol), water (21 μL, 10% wt), and a PTFE milling ball (16 mm, 4.40 g), sealed in 25 mL vessels, and milled for 60 min at 30 Hz. Then, ^1^HNMR was prepared according to the methodology described in Section 2.4.

## 3 Results and Discussion

### 3.1 Kinetics as a Function of Milling Frequency and Ball Diameter

To ensure repeatability of the kinetics across instruments and institutions, we began by replicating the results of Hutchings et al. using 15 mm milling balls in SS jars. The reaction shows the expected sigmoidal kinetics, with earlier feedback period onset correlated to higher milling frequencies (see Figure 2a).^32^ The reagent mixture rheology changes throughout the milling process, with the crude mixture either coating the milling ball or notably coating the vessel walls (see Section 5 of the Supporting Information). When removed, the coating remains hardened in a stable shape for up to 2 weeks, contrary to literature reports.^32^ These initial findings are inconsistent with the phenomenological model previously proposed, where powder condenses into a hard solid film around the ball during the positive feedback period, increasing reaction rate via an increased number of effective collisions.^33^ This effect would be unlikely given inconsistent snowballing and reports where reagent caking and insufficient mixing are associated with reduced reactivity.^42–48^ To explore further, we decreased the milling ball size to 12.7 mm to form a thicker reagent layer coating on the ball surface during the feedback period. Snowball formation remained inconsistent at 30 Hz and did not occur at 25 Hz (see Figure 3). Smaller milling media also led to a slower onset of the feedback period and an associated slower kinetic acceleration within the sigmoidal curve, as shown by the visual “flattening” (see Figure 2b).

**Figure 2:**
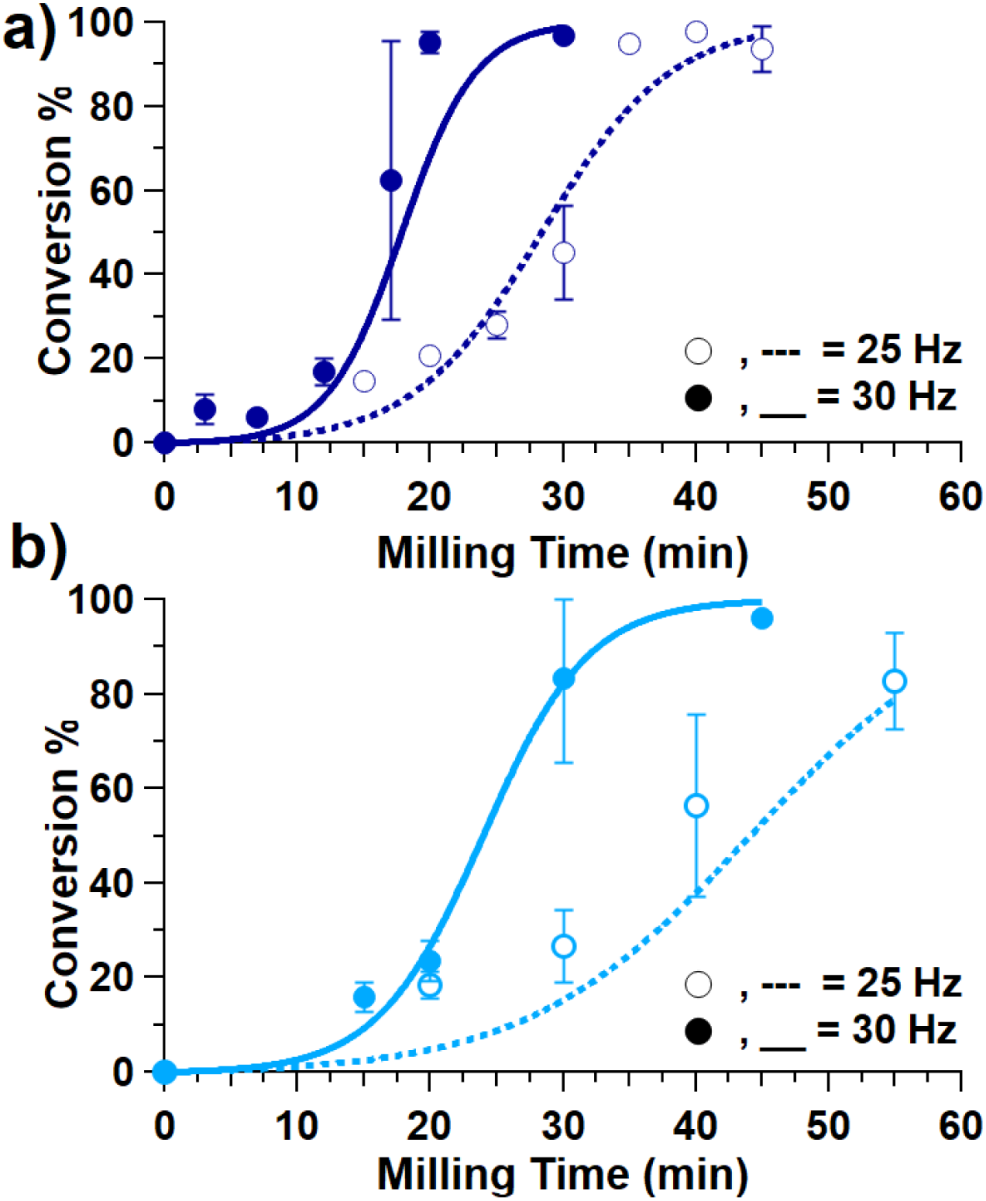
Reaction kinetics in SS at 25 and 30 Hz using: a) a 15 mm SS ball and b) a 12.7 mm SS ball. All curves shown were replicated by four trials, and error is given as the standard deviation. Curve fits shown are taken from the autocatalytic model described below in Figure 4. Regression statistics are not shown in the curves but can be found in the Supporting Information Section 9.

**Figure 3:**
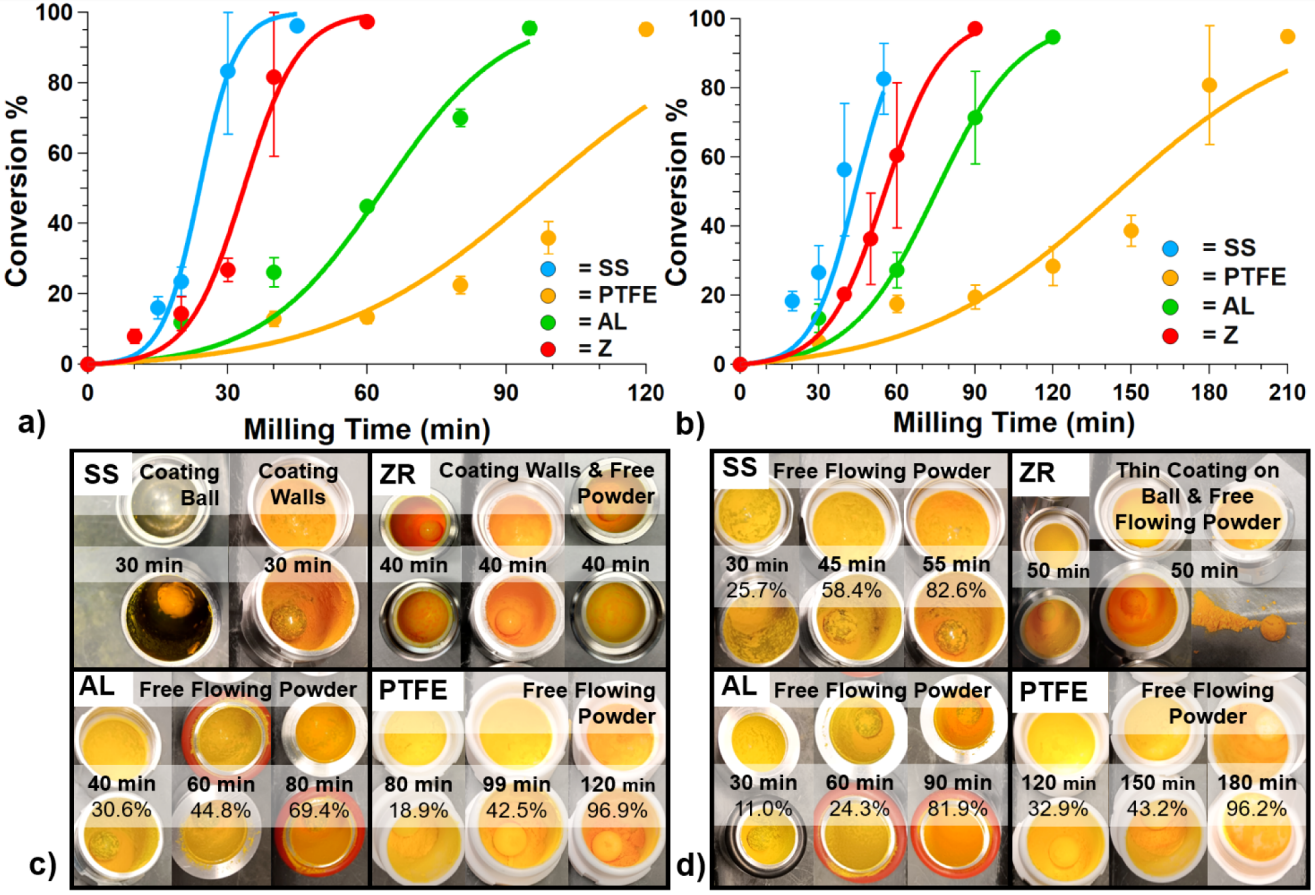
Reaction kinetics in SS, ZR, AL, and PTFE at a) 30 Hz and b) 25 Hz and reaction product rheology in SS, ZR, AL, and PTFE at c) 30 Hz and d) 25 Hz during the feedback period. Reaction conditions were as follows: scale (1.9 mmol, 10 wt % H_2_O), jar size (25 mL), and ball size (12.7 mm). Curve fits shown are taken from the autocatalytic model described below in Figure 4. Regression statistics are not shown in the curves but can be found in the Supporting Information Section 9.

Surprisingly, feedback was observed to begin when the mixture reached about 20% conversion, regardless of frequency or milling ball size (see Section 5 of the Supporting Information). This explains why others have not observed a similar feedback period at 15 Hz, as the kinetics of the reaction were tracked only to 20% conversion.^32^ The current phenomenological model is unable to account for these findings and other reports where rheological changes were not observed using multiple milling balls in both planetary and vibratory mills.^28,31^

### 3.2 Kinetics as a Function of Milling Jar Material and Force

To evaluate the effect of kinetic energy, adhesion to the surface, and material hardness on reactivity, we examined the kinetics of the reaction in a variety of different milling materials with a wide range of mechanical hardness at both 25 and 30 Hz. For consistency, these trials used the same reaction conditions and methodology, employing available 12.7 mm milling balls for all materials. The results of these studies are summarized in Figure 3, and further milling material details can be found in Section 2 of the Supporting Information. Attempts to fit the kinetics to classical first- and second-order kinetic models failed for all conditions (see Section 6 of the Supporting Information). Thus, consistent with previous literature reports, a positive feedback process dominates the transformation under mechanochemical conditions.^32^ The fact that this process occurs regardless of the material choice indicates that a catalytic effect due to the milling medium is not responsible for the enhanced rate during the feedback period.

Decreasing reaction frequency results in decreased reaction rates for all materials studied. A variation in kinetic acceleration, akin to that observed when the frequency is changed, is also seen between different milling reactor materials with rates being highest in SS, followed by ZR, AL, and finally PTFE (see Figure 3). One important observation is that reaction rates do not cleanly correlate to material elastic modulus or Rockwell Hardness, as although ZR is ∼7% stiffer than SS, the reaction rates are notably lower than SS. This occurs because the impact force in a ball mill depends strongly on the amount of kinetic energy of the milling ball (see Section 7 of the Supporting Information for a derivation of impact force as a function of the material mechanical properties). If we presume that the milling ball velocity is set constant by the frequency of the shaker mill, then the kinetic energy scales with the material density for balls of identical size. Table 1 summarizes the material properties of the milling balls (elastic modulus, E; Rockwell B Hardness; and density) used here with a relative measure of their kinetic energy under identical milling conditions.

**Table 1:** Material Properties of the Milling Balls and Associated Kinetic Energies

Material	E (GPa)	Rockwell B Hardness	Density (kg/m^3^)	Relative Kinetic Energy
**SS**	193	70	7900	1.00
**ZR**	206	80	5810	0.74
**AL**	69	60	2700	0.34
**PTFE**	0.5	25	2130	0.27

Significantly, the snowball effect (when observed) was only found in SS and ZR jars. ZR snowball formation appears inconsistent (as seen with SS, see Section 3.1), with the snowball only observed in a single trial after 40 min of milling at 25 Hz. The layer of product coating the zirconia ball was much thinner, with a greater portion of the powder remaining free-flowing, likely due to lower adhesive forces between the reagents and the ZR surface. On all other occasions (e.g., PTFE and AL), the material appeared to favor remaining as free-flowing powder.

The onset of the positive feedback period observed and the conversion % reached at that point in each material are shown in Table 2. Mimicking the findings in SS, the feedback period begins once the conversion reaches about 20%. The length of time before the positive feedback period occurs similarly seems to relate to the mechanical properties, with longer times seen at lower frequencies or with softer materials. This evidence suggests that rheological factors are less critical to obtaining the sigmoidal kinetics than previous literature data indicated.^32,33^

**Table 2:** Onset of Kinetic Feedback Period as a Function of Milling Frequency and Time, and % Conversion at That Point

	Feedback Onset (min)		% Conversion	
Material	25 Hz	30 Hz	25 Hz	30 Hz
**SS**	33.4	18.6	21.3	20.4
**ZR**	41.2	25.9	21.2	21.6
**AL**	53.0	46.1	19.9	20.5
**PTFE**	93.5	67.7	19.5	20.6

To further validate this assertion that the autocatalysis is facilitated by the initial conversion of reagents to product, we intentionally introduced 20% of the product into the reaction systems for SS and PTFE (using the standard jar sizes), with the starting ratios of vanillin:barbituric acid:product of 8:8:2, and found that in SS, the induction period was reduced to below 10 min, and in PTFE to below 50 min, thus clearly indicating the autocatalytic behavior (see Supporting Information Section 11), suggesting that a critical precursor state is needed.

### 3.3 Modeling the Reaction Kinetics

Recent work by Lukin et al. showed that the reagents can undergo transformation into a cocrystal intermediate, wherein reagents are suggested to be optimally oriented within the lattice to enable further condensation.^31^ We have carried out a few precursory experiments on reagent powders to explore this cocrystal and isolate its reactivity. Vivid yellow cocrystal formation (validated by PXRD) occurs readily in the solid state via vanillin diffusion preferentially into the barbituric acid when the powders are placed together, touching either in dry forms or after separate milling with 10 wt % water (see Section 8 of the Supporting Information). This suggests that the cocrystal does not require milling to form in the presence of water, in contrast to the significant barriers observed when milling in other liquids, such as strong non-nucleophilic bases like *N*,*N*-diisopropylethylamine.^31^ Moreover, cocrystal formation is found to be near instantaneous when reagent powders are simply shaken together (by hand ∼15–25 s) in the presence of 10 wt % water (regardless of premilling), and is accelerated at higher temperatures or in the presence of excess water (see Section 8 of the Supporting Information). Product formation was found to follow cocrystal formation, with the reaction proceeding slowly at low temperatures and rapidly when the cocrystal was placed on a 70 °C hot plate (see Section 8 of the Supporting Information). At high temperatures, product formation may proceed through a eutectic intermediate, as previously proposed, given the rapid reaction rate and darker product coloration (see Section 8 of the Supporting Information).^31^ Excess water was also observed to accelerate the formation of the product from the cocrystal at both low and high temperatures (see Section 8 of the Supporting Information). Autocatalysis by existing reagent powder was also tested. We observed that, at high temperatures, the product from cocrystal will preferentially form near existing product (see Section 8 of the Supporting Information); similarly, cocrystal formation at higher temperatures may be favored by existing product but is harder to demonstrate definitively due to the accelerated rate of transformation (see Section 8 of the Supporting Information).

Working off these findings, we offer a simple autocatalytic model for our kinetic measurements. Initially, the cocrystal must form with a rate constant of *k*_*C*_ while product formation follows cocrystal formation with a rate constant of *k*_*P*_, as shown in Figure 4. The rate of cocrystal formation is fast when reagents mix in the presence of water, while evidence suggests the rate of transformation from cocrystal to product is also rapid when the cocrystal is heated to temperatures typically reached in the ball-milling process.^32^ Assuming one of these rates is quite rapid, or the rates are equivalently fast, we can simplify the reaction equation to

Reaction 1 $$2A\to P+H_{2}O$$

**Figure 4:**
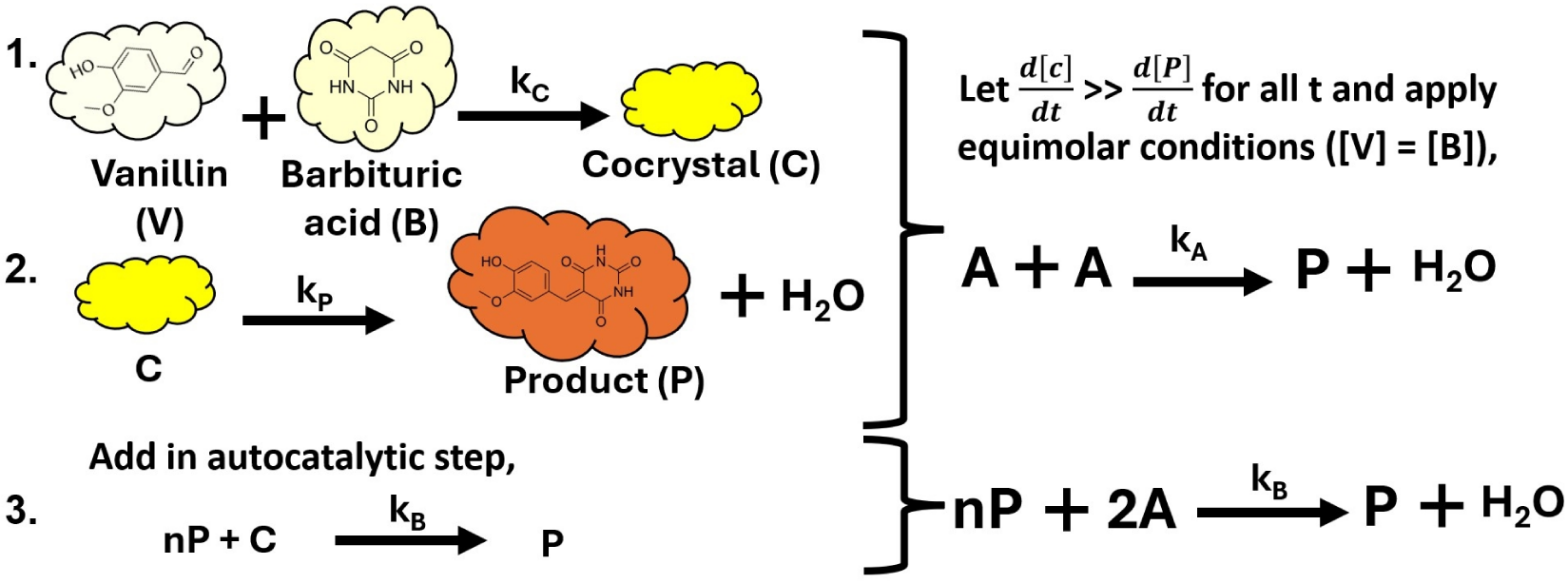
Proposed steps in the mechanochemically driven condensation of vanillin and barbituric acid (left) and the simplification of the reaction for the autocatalytic model used to fit the kinetics (right), where A refers to the amount of a single reactant.

with a rate constant of *k*_*A*_ as shown in Figure 4, where *A* represents the amount of a single reactant (a simplification of the mathematics taking advantage of the fact that vanillin and barbituric acid are added to the jar in equimolar amounts and react in a 1:1 stoichiometry) and *P* is the amount of product formed. This assumption was verified by testing kinetic models incorporating the cocrystal intermediate, which failed to fit the data more accurately than this simplified system. Subsequently, we add the assumption that the rapid acceleration in the observed reaction rate is caused by autocatalysis, where

Reaction 2 $$2A+nP\to P+H_{2}O$$

with a rate constant of *k*_B_ as observed in cocrystal experiments (see Figure 4). This results in a model of the kinetics of the reaction using the following equations:

1 $$\frac{dA}{dt}=-2k_{A}A-2k_{B}AP^{n}$$

and,

2 $$\frac{dP}{dt}=2k_{A}A+2k_{B}AP^{n}$$

where n is the degree of autocatalysis.^49^

To avoid overfitting the data set due to the limited number of points that could be obtained by ex-situ measurements, we assumed that *n* = 1 (the quality of the regression was not strongly dependent on reaction order, see Section 9 of the Supporting Information for regression analysis details), and we fixed *k*_A_ = 1×10^‑5^ *s* ^‑1^ to determine if the remaining rate has a dependence on the elastic modulus of the milling media, the frequency of milling, and the weight of the ball. The rate *k*_B_ was chosen as the fitting parameter because the slope of the reaction at short times (when *k*_*A*_ is dominant and product concentration is building up before autocatalysis occurs) was found to be weakly dependent on the experimental parameters (being consistent across frequencies and materials via an initial rates analysis using early points) and was seen to fail to fit the data well independently in all cases. Solutions to eqs 3 and 4 and were numerically regressed to the data to provide associated reaction fits shown in Figures 2 and 3 (see also Section 9 of the Supporting Information).

Figure 5 shows the reaction rate constants *k*_B_ resulting from a regression with eqs 3 and 4 for each trial (see Section 9 of the Supporting Information). The results show that the reaction rate constants are strongly dependent on the elastic modulus of the material, the milling frequency, and the weight of the milling material. All of the parameters have an expected positive correlation with impact force^50^ and suggest that this reaction is mechanically driven. Note that all trials exhibited autocatalytic behavior, regardless of the presence of a reactant film on the milling media or the material type.

**Figure 5:**
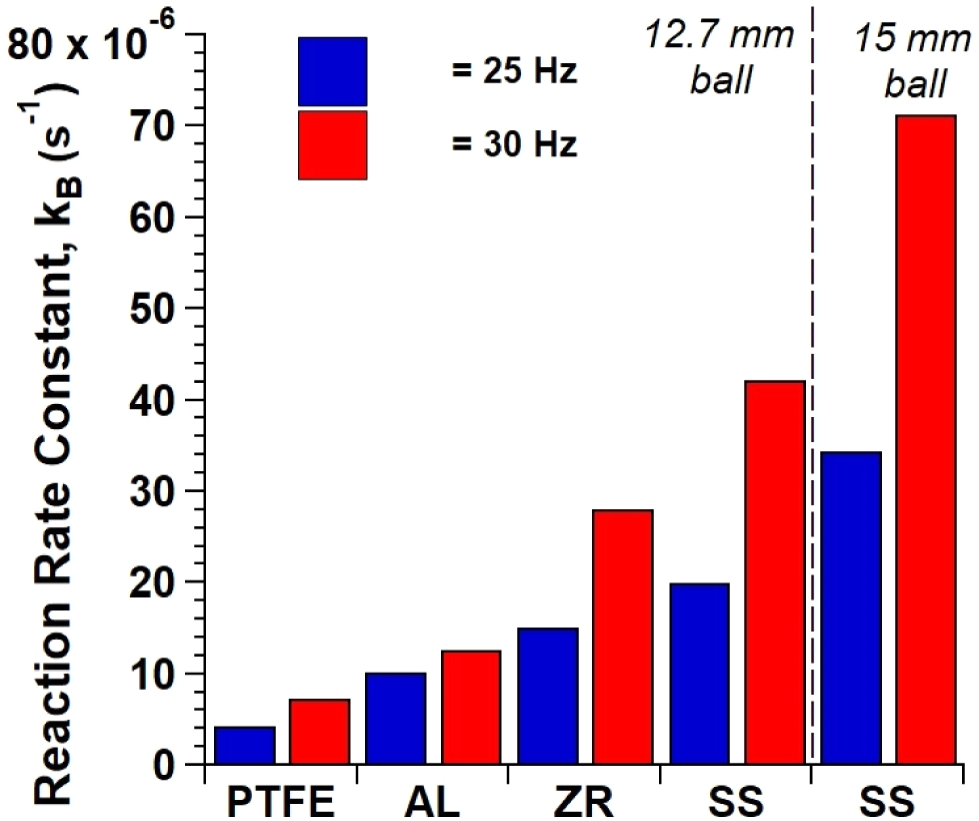
Experimental reaction rate constant (k_B_) showing the dependence of the reaction on mechanical properties.

Utilizing these experimental fits, we investigate whether the autocatalytic reaction is mechanically activated. Consider a reciprocating ball mill that translates a distance *L* with a frequency of *f*. If we assume that, on average, the ball within the mill has a speed of zero relative to the global frame of reference and a speed *v* = *fL* with respect to the reciprocating mill, then the ball impacts the vessel with an energy *K* = 0.5*mv* ^2^where *m* is the ball’s mass. The local product formation rate density at any location and time (*t*) *within a single impact* takes the form:

3 $$\frac{d\varphi_{2}\left(r,t\right)}{dt}=2k_{1}\varphi_{1}\left(r,t\right)+2k_{2}\left(r,t\right)\varphi_{1}\left(r,t\right)\varphi_{2}{\left(r,t\right)}^{n}$$

where *φ* is the local reactant concentration (*mol*/*m* ^3^) for each species (with the subscript of 1 corresponding to *A* and the subscript of 2 corresponding to *P* from eqs 3 and 4 , *r* is the radial position from the center of the impact zone, and *n* is the reaction order. For more information on this approach, consult Nwoye et al.’s work to correlate force and reaction rates in vibratory mills.^50^

If we assume that the reaction within the impact zone can be approximated using an *average* impact pressure and an *average* reactant concentration within a cylinder of material with some unknown height *h* and an impact radius of *a*, the rate equation is multiplied by the impact reactant volume and becomes only a function of time during the impact,

4 $$\frac{dN_{2}\left(t\right)}{dt}=\pi a^{2}h\left[2k_{1}\varphi_{1}\left(t\right)+2k_{2}\varphi_{1}\left(t\right)\varphi_{2}{\left(t\right)}^{n}\right]$$

where *N* is the number of molecules reacted *during the impact*. Converting between eqs 5 and 6 can be thought of as averaging out spatial effects within the impact zone. If we further assume that the reaction proceeds slowly within a single impact (valid for reactions that require many thousands of impacts to complete), then the change of reacted molecules within the impact becomes

5 $$\Delta N_{2}\left(t\right)=\pi a^{2}ht_{tot}\left[2k_{1}\varphi_{1}\left(t\right)+2k_{2}\varphi_{1}\left(t\right)\varphi_{2}{\left(t\right)}^{n}\right]$$

where *t*_*tot*_ is the total time for a *single* impact. Note that all *φ*(*t*) are based on how the whole system within the reactor is changing, while Δ*N*_2_(*t*) focuses on the number of product molecules created within a single impact. Thus, the generation of the product over many impacts in time must be calculated by multiplying eq 7 by 2*f*, the number of impacts per second.

6 $$\frac{dN_{2}\left(t\right)}{dt}=2f\pi a^{2}ht_{tot}\left[2k_{1}\varphi_{1}\left(t\right)+2k_{2}\varphi_{1}\left(t\right)\varphi_{2}{\left(t\right)}^{n}\right]$$

Equation 8 provides the number of product molecules made in the impact events, but we need to convert to the number of molecules in the whole jar because the density functions change as a result of the impact events. Thus, *dN* is converted back to molecular density by assuming that the reacted molecules from the impact are perfectly mixed with the reactants in the vessel before undergoing another impact, and recognizing that Δ*φ* = Δ*N*/*V*, where *V* is the total volume of material in the reactor jar. This manipulation enables the model to scale appropriately from the macroscale view of the transformation within the reactive contact to an atomic-scale transition state theory, generating:

7 $$\frac{d\varphi_{2\left(t\right)}}{dt}=\frac{2f\pi a^{2}ht_{tot}}{V}\left[2k_{1}\varphi_{1}\left(t\right)+2k_{2}\varphi_{1}\left(t\right)\varphi_{2}{\left(t\right)}^{n}\right]$$

Note that this equation can be generalized to reactions of any form by simply modifying the rate terms within the square brackets (according to any proposed reaction mechanism and associated stoichiometry), and has been solved previously for standard single-term reaction rates for any reaction order.^50^ We can see that the reaction rate constant *k*_B_ from eq 4 can be related to *k*_2_ as shown in eq 10

8 $$k_{B}=\frac{2f\pi a^{2}ht_{tot}}{V}k_{2}$$

Assuming elastic collisions, Hertz’s theory of impact provides estimates for *a* and *t*_*tot*_, where *a* written in terms of the impact energy, is given in eq 11,

9 $$a={\left(\frac{3KR^{2}}{4E_{s}}\right)}^{1/5}$$

where *R* is the radius of the impact, *K* is the kinetic energy, and *E*_*s*_ is the effective mechanical modulus. Using the elastic modulus, *E* and Poisson ratio, *γ* for the ball and vessel materials, the effective mechanical modulus *E*_*s*_is given in eq 12:

10 $$E_{s}={[\frac{1-\gamma_{1}}{E_{1}}+\frac{1-\gamma_{2}}{E_{2}}]}^{-1}$$

The radius of impact (*R*) is expressed in terms of the radius of the ball (*R*_*b*_) and the radius of the reaction vessel’s curved ends (*R*_*c*_) as given in eq 13:

11 $$R={\left({\frac{1}{R}}_{b}-{\frac{1}{R}}_{c}\right)}^{-1}$$

The expression for impact time in Hertz theory is given by eq 14:^51^

12 $$t_{tot}=2.8683{\left(\frac{m^{2}}{R\gamma E_{s}^{2}}\right)}^{1/5}$$

The rate constant for the reaction can thus be proportionally related to the observed rate constant over time in the ball mill as (per second),

13 $$k_{rxn}=6.747\frac{\pi hR^{3/5}}{VLE_{s}^{4/5}}k_{eff}K^{4/5}$$

which deconvolutes the force-dependent volume of reactants within each impact from the force-accelerated thermochemical rate constant. Note that this “reactant volume” depends on the size of the ball, the hardness of the ball and vessel, the length (*L*)) of the mill, and the kinetic energy (*K*) of the ball, which depends on ball mass and shaking frequency. The value of *k*_*eff*_ can be modeled using a thermal activation model as in eq 16:

14 $$k_{eff}=k_{o}e^{-\frac{E\left(P_{av}\right)}{k_{b}T}}\approx k_{o}e^{-\frac{E_{a}-P_{av}\Delta V_{a}}{k_{b}T}}$$

where *k*_*o*_ is an attempt frequency (typically on the order of 10^12^ – 10^15^ *s* ^–1^ for solid state reactions), *E* is the activation energy of the reaction, *P*_*av*_ is the average pressure in the contact at maximum indentation, *k*_*b*_ is Boltzmann’s constant, and *T* is the temperature. This general form of the energy barrier is typically assumed to evolve linearly with applied pressure, where the thermochemical barrier is *E*_a_ and the mechanical work done on the reaction is given by multiplying pressure by a change in activation volume Δ*V*_a_. Taking the natural log of both sides results in eq 17

15 $$\ln\left(k_{eff}\right)=\ln\left(k_{o}\right)-\frac{E\left(P_{av}\right)}{k_{b}T}$$

Thus, the magnitude of the energy barrier is proportional to the negative natural log of the reaction rate *E* ∝ −In­(*k*_*eff*_).

Here, we note that the contact pressure scales with frequency as *P* ∝ *f* ^2/3^, and so reaction rate constants in any ball milling reaction scale with frequency as

16a $$k=\alpha f^{8/5}e^{\beta f^{2/3}}$$

16b $$\alpha =38.29\left(\frac{h}{V}\right)\left(\frac{R^{3/5}R_{b}^{12/5}\rho_{b}^{4/5}}{E_{s}^{4/5}}\right)L^{3/5}k_{o}e^{-\frac{E_{a}}{k_{b}T}}$$

16c $$\beta =0.512\left(\frac{E_{s}^{4/5}R_{b}\rho_{b}^{1/3}}{R^{3/5}}\right)\frac{\Delta V_{a}}{k_{b}T}$$

where *α* and *β* are constants with values that depend on the milling materials, milling conditions, and dynamic stiffness of the molecules under load. This equation unifies the many disparate frequency dependencies in the literature, where weakly force-accelerated activated reactions observed over a small change in frequency appear to be nearly linear within experimental uncertainty (*f* ^8/5^), and where strongly force-accelerated reactions appear to follow a power law in frequency with larger powers.^52,53^ Power law regression of eq 18 shows R^2^ > 0.999 for powers up to 7. Most of the terms in this equation are readily measured for the reactor conditions chosen, except for *h*, *E*_a_, and Δ*V*_a_. Estimating the powder film thickness to be ∼50 μm (roughly the size of reactant powders), the values of Δ*V*_a_ for energy barriers *E*_a_ ∼30 kcal/mol are on the order of tens of cubic angstroms for the reaction studied here, consistent with other studies of atomic-scale mechanochemical reactions.^54–56^ A more precise determination of the activation volume could be obtained by systematically measuring reaction rate as a function of both frequency and temperature.

The trend of the energy barrier as a function of impact pressure provides additional physical insights into the chemical system under study. Figure 6 shows a plot of the average contact pressure vs the calculated normalized energy barrier, where the barrier is large at low impact pressures and quickly reduces as the pressure increases, until the barrier essentially vanishes. At this point, the reaction is limited by the size of the contact made between the ball and the vessel wall during the impact. This reduction in the energy barrier with force is evidence that the reaction between vanillin and barbituric acid is a force-accelerated autocatalytic process. The present example demonstrates that the energy barrier decreases nonlinearly with applied pressure at large loads, consistent with previous mechanochemical studies of the Hammond Effect, which found evidence of this behavior in other chemical systems.^57,58^ The plot suggests that the energy barrier change is negligible for pressures above ∼1.0 GPa, which means further increases in impact load provide negligible benefit to the reaction rate, and likely increase energy losses, dissipated as excess heat. This result suggests that the mechanical efficiency of a ball mill reactor decreases with increasing force for the reaction studied here.

**Figure 6:**
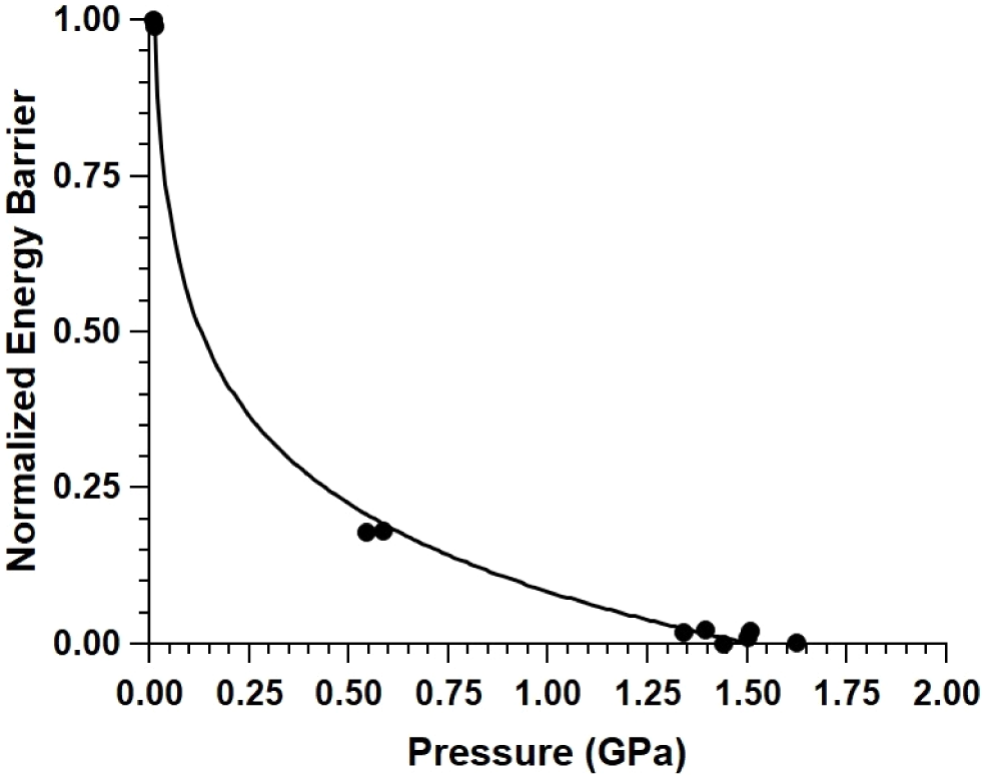
Graph of normalized activation energy barriers calculated from experimental rates as a function of pressure experienced in the reactor based on mechanical properties. Logarithmic fit is given to guide the eye.

### 3.4 Reaction Progression in Different Jar Regions Subject to Primarily Shear or Impact Forces

The reaction mixture of vanillin and barbituric acid showed the capacity to adhere strongly to SS, weakly to ZR, and weakly to AL, with little to no preference for PTFE. Exploiting this behavior offered a unique opportunity to observe which regions in the ball mill were most useful for driving the reaction. To achieve this, we designed and machined a system in-house to segment the ball-mill jar into areas made of different materials.^40^ The system consists of jar inserts made with internal dimensions matching a standard 10 mL Retsch MM 400, designed to nest snugly within standard Retsch MM 400 25 mL jars (shown in Figure 7 and described in greater detail in Section 2 of the Supporting Information).

**Figure 7:**
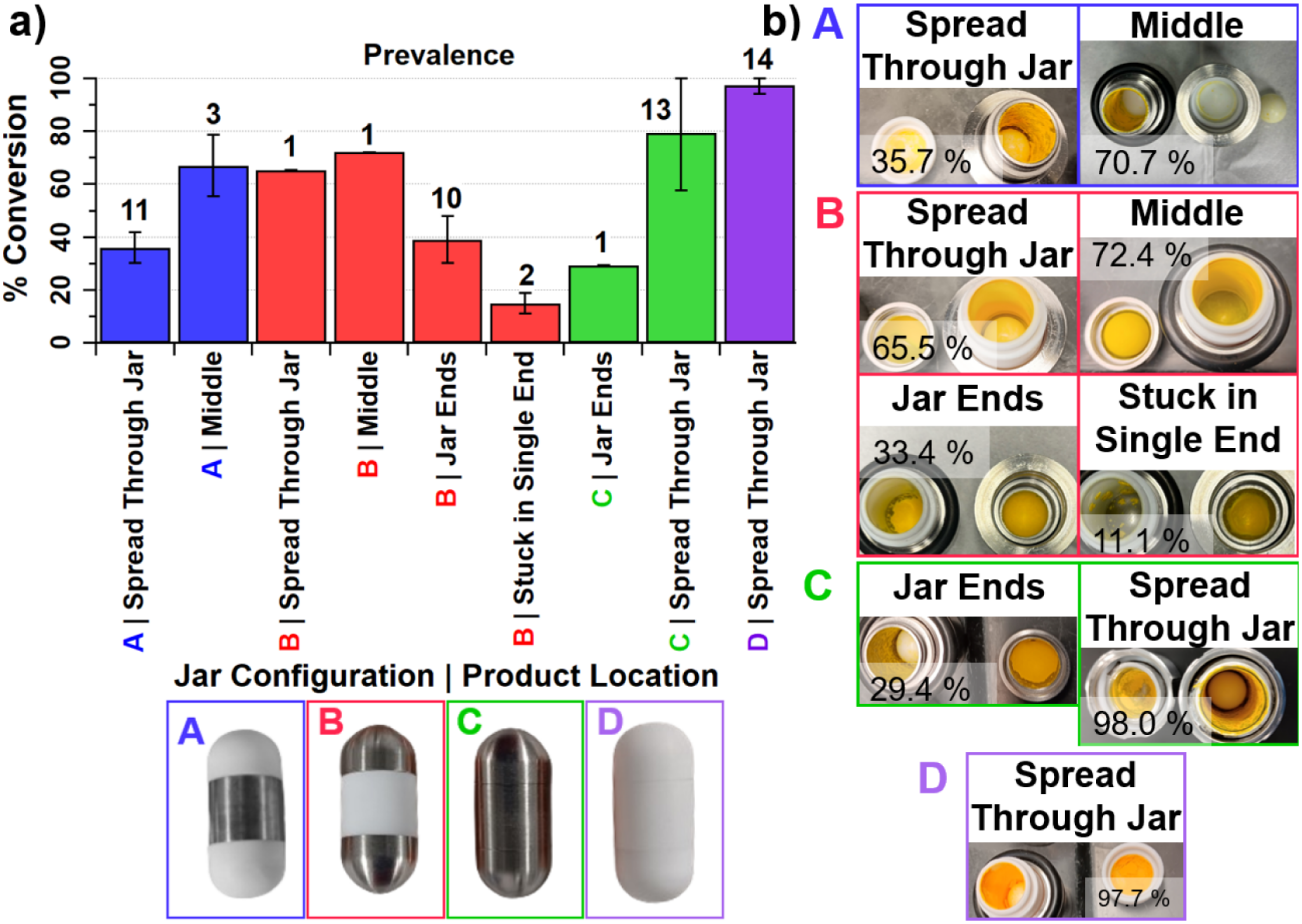
a) Different reaction conversions after 60 min of milling at 30 Hz (PTFE ball, 16 mm, 4.4 g) correlated to the milling materials and their respective final stuck locations within the 10 mL milling vessel inserts. The number of trials with the observed product in the jar region identified are provided above the bars, showing average % conversion, while the standard deviations of the trials are shown at the tops of the bars. b) Images of the reagent mixture adhered to different regions of the milling jar.

We designed our custom jars to place PTFE and SS strategically along either the end or middle of the jar, hypothesizing the material would preferentially stick and react either at the ends of the jar or in the middle. We then utilized a large PTFE milling ball, which fit with ∼2 mm of clearance on either end, to ensure that the middle of the jar experienced solely shear forces, while impact forces were confined to the ends of the jar. With this setup, we could examine whether shear or impact forces dominated in driving the transformation in situ within commercial ball milling instrumentation.^59,60^

Experiments were performed according to the methodology described in Section 2.5. The reaction yield in the 10 mL pure PTFE vessel (case D) shows that the reaction mixture is thoroughly spread out within the jar upon completion due to the poor adhesion of the chemical reagents to the vessel surface. The high mobility of the reaction powder correlates with complete conversion %, suggesting enhanced mixing of the reactants within the jar when adhesion is minimized (see Figure 7). In this control case, both shear and impact forces occur, and it cannot be determined which dominates because of the homogeneity of the milling jar environment. Using this as a benchmark, we then explored the reaction in the 10 mL SS vessel (case C); here, the reaction shows a lower conversion of 80% and less consistency (SD = 20) in reaction performance when reagents spread through the jar (see Figure 7). This behavior suggests that greater adhesion to the SS vessel throughout milling decreases reaction mixing and thus rates compared to the PTFE case. A comparatively poor conversion of 29.4% occurred in one trial where reagent was packed exclusively at the ends of the milling jar; the decrease could be due to either reduced mixing within the jar due to impaction at the jar ends or due to the lack of shearing, which occurs primarily in the center of the vessel (see Figure 7).

Yields were then studied for the case where the ends were PTFE and the center was SS (case A), or where the ends were SS and the center was PTFE (case B). For case A, the reagents proceed to either: 1) stick exclusively to the SS in the middle, or 2) spread throughout the jar (Figure 7, setup A). In case 1, reagents predominantly undergo shear forces once the powder adheres as the ball slides across the trapped reagent. This resulted in surprisingly high yields of 72.1%. However, this result is still lower than the yield with pure PTFE jars, suggesting a mobility loss associated with reagent confinement. In case 2, the conversion % remains low at 36.0%, matching the yields observed in pure SS, where reagents appeared to aggregate at the ends of the jar. This suggests that while reagents do not end up permanently stuck at the ends of the jar, they likely still stick on occasion through the milling process. This results in the reagents undergoing more impact forces than shear forces over time and generally having less mobility (as evidenced by the reduced yield compared with case 2).

Upon isolating PTFE to the middle of the jar (case B), the product is found to predominantly stick to the SS ends (Figure 7, setup B). This results in slightly higher conversions than that observed in pure SS jars of 39%, suggesting that the slippery middle of PTFE may act to slightly enhance reagent mixing compared to the SS jar middle, even though reagents end up confined to the impact regions. This is likely a result of reduced reaction forces when the reagent layer is thicker. On one rare occasion, the reagent was observed to stick exclusively to the PTFE in the middle of the jar, resulting in high reaction conversion at 72.4%. This may suggest a preference for the reaction to be driven by shear forces over impact forces. Within error, this result also matches the high conversion of 65.5% observed when the reagent remains free-flowing (or sticks and unsticks).

These higher yields indicate that the location of slippery PTFE in the middle of the jar can be exploited to facilitate better mixing than when the entire jar is made of SS. In this setup, the presence of SS at the ends of the jar always results in some hampering of reagent movement during the milling process, as the yield is lower than when performed in PTFE alone. When reagents nest at the ends of the jar, the reaction is always less effective than when reagents can move in larger portions of the jar. This is likely due, in part, to the thicker packing of the reagent reducing energy transfer through the matrix when reagents are located at the ends, rather than the thinner layer observed when the reagent is spread over the middle jar portion. This increased shearing and spreading of materials in these smaller volume jars results in faster overall conversion, as compared to the results shown in Figure 3.

Future work will involve designing larger jars to correlate with the jar sizes used in the kinetic measurements, exploring the effect of varying jar loading, and investigating the impact of reducing the ball size to allow for greater ball mobility. Significantly, this work demonstrates that mixing and reagent film breakdown, as opposed to formation, are critical in maintaining high reaction rates. In all conditions observed, reagent agglomeration into thick films and confinement were correlated with the lowest observed yields, even if reagents were located directly at jar ends, where they were subjected to the highest impact forces and a greater number of impacts. While partially contradictory to previous claims that reagents forming films around the milling media to experience more impacts ought to enhance reaction rates,^33^ our work here offers a broader perspective on how changing the material used for the milling jars impacts the distribution of reactant materials as films through-out the reaction chamber to better facilitate transformation to cocrystals and then onto products via shear thinning. It is the shear thinning that also likely increased yields in prior work when a snowball was formed on the milling ball. Clumping seems to generally result in poorer mixing and reduced reactivity, as observed in many other mechanochemical systems.^42–48^ A further critical study to undertake will now be to examine the role of the product in facilitating autocatalysis and how the interfacial interactions between the milling jar surfaces, reagents, cocrystals, and product conspire to support that. This will enable us to then extend this work to other Knoevenagel condensation reactions to explore whether similar kinetics are seen.

## 4 Conclusion

We have measured the kinetics of the reaction between vanillin and barbituric acid in various milling materials and demonstrated that rheology alone cannot explain the positive feedback period characteristic of the mechanochemical reaction. Instead, we posit that the formation of a cocrystal film, which undergoes shear flow, then yields a force-accelerated autocatalytic process. Such shear flow conditions are likely what have given rise to previous reports of a “cohesive state” or “snowball effect,” but more aptly describe the systems in question. These findings are supported by a new methodology we introduced in this work, using multicomponent milling jars, that allow for the investigation of the mixing and role of different jar regions and forces experienced during the reaction in commercial milling systems. With this approach, we showed that when using thin, small vessels with confined milling ball movement, shear forces and effectively reduced friction are critical for reaction progress, while normal impact force alone often leads to densely packed films that inhibit full conversion. This multicomponent milling methodology can be broadly applied to a host of reaction systems for facilitating in a deeper understanding of how mechanochemical milling environments, and the types of forces at play, alter mechanochemical reaction outcomes.

## Supplementary Material



## Notes

Chemicals and hazards, materials and methods, general synthetic procedures, additional reaction mixture rheological data, kinetics fitting with first order and second order models, experiments with diffusion driven reactivity, regression analysis for kinetic fits, modeling impact force in a vibratory ball mill (PDF)

## Abbreviations

SS: stainless steel
PTFE: polytetrafluoroethylene
AL: aluminum
ZR: zirconia
NMR: nuclear magnetic resonance
PXRD: powder X-ray diffraction
