# Supplementary material for: Force-Accelerated Autocatalysis in a Knoevenagel Condensation Reaction during Ball Milling
Source: J Am Chem Soc. 2026 Jul 10;148(28):29697–709. doi: 10.1021/jacs.5c23405 (PMC13397893; doi:10.1021/jacs.5c23405)
Supplement: Supplementary file 1 [file ja5c23405_si_001.pdf]

SUPPORTING INFORMATION

Force-Accelerated Autocatalysis in a Knoevenagel Condensation Reaction During Ball Milling

Kathleen R. Floyd,[1] Emmanuel C. Nwoye,[1,2] Lizette Mella,[1] Paulina V. Gonzalez,[1,4] Jonathan R. Felts,[2*] and James D. Batteas[1,3*]

1. Department of Chemistry, Texas A&M University, College Station, TX 77842-3012, USA. Email: batteas@chem.tamu.edu
2. Advanced Nanomanufacturing Laboratory, Department of Mechanical Engineering, Texas A&M University, College Station, Texas-77843-3123, USA. Email: jonathan.felts@tamu.edu
3. Department of Materials Science and Engineering, Texas A&M University, College Station, TX 77842-3012, USA.
4. Department of Chemistry, The University of Texas at Austin, Austin, TX 78712-1224, USA.

TABLE OF CONTENTS

1. CHEMICALS AND HAZARDS 3

2. MATERIALS AND METHODS 3

2.1 Milling Jar Material Specifications 3

2.1.1 Material Suppliers & Milling Jar Manufacturing 3

2.1.2 Milling Ball Sizes and Weights 4

2.1.3 System for Putting Different Materials in Different Milling Jar Regions 5

2.2 NMR 5

2.2.1 Instrumentation 5

2.2.2 NMR Reference Data 5

2.3 PXRD 6

2.4 Temperature measurements 6

2.5 Autocatalysis experiments 7

3. GENERAL PROCEDURES FOR 5-(4-HYDROXY-3-METHOXYBENZYLIDENE)PYRIMIDINE-2,4,6(1H,3H,5H)-TRIONE SYNTHESIS 7

3.1 Experiments Tracking the Kinetics in Different Materials 7

3.2 A Note on Reaction Progression After Milling in the Solid 8

3.3 A Note on Reaction Progression in Solution 8

4. DETERMINING THE ONSET OF THE FEEDBACK PERIOD: 1ST and 2ND DERIVATIVES OF REACTION KINETICS FITS 8

5. RHEOLOGY IN STAINLESS STEEL AS A FUNCTION OF FREQUENCY AND BALL SIZE 12

6. FITTING THE KINETICS TO TRADITIONAL MODELS 14

7. IMPACT FORCE MODEL IN A VIBRATORY BALL MILL 15

8. EXPERIMENTS WITH DIFFUSION DRIVEN REACTIVITY 17

8.1 Interactions Between Dry Reagents 17

8.2 Interactions Between Pre-Milled Wet Reagents 18

8.3 Interactions Between Wet Reagents (Without Pre-Milling) 19

8.4 Experiments Testing Product Autocatalysis 21

8.5 PXRD to Identify the Yellow-Colored Compound 23

9. REGRESSION ANALYSIS FOR KINETIC FITS 23

10. TEMPERATURE MEASUREMENTS 25

11. AUTOCATALYSIS EXPERIMENTS 28

12. DOI LINK TO RAW DATA FILES 29

13. REFERENCES 29

1. CHEMICALS AND HAZARDS

Reagents are commercially available. For each substance, hazards are indicated according to Regulation (EC) No 1272/2008.

vanillin: CAS [121-33-5], 99% BeanTown Chemical, Lot #50084050 – H317, H319

- vanillin can cause allergic skin reaction and serious eye irritation

barbituric acid: CAS [67-52-7], 99% BeanTown Chemical, Lot #50070292 – H315, H319, H335

- barbituric acid can cause skin, eye, and respiratory irritation

water: water was purified in house using a Barnstead Nanopure system (18.7MΩ・cm)

2. MATERIALS AND METHODS

2.1 Milling Jar Material Specifications

2.1.1 Material Suppliers & Milling Jar Manufacturing

Trials employed stainless steel milling jars (25 mL) and associated stainless steel grinding balls (15 mm) purchased from Retsch along with grinding balls (12.7 mm) made of corrosion resistant 316 stainless steel purchased from McMaster Carr. Zirconia jars (25 mL) were made by Retsch and zirconia grinding balls (12.7 mm) were purchased from McMaster Carr. PTFE (25 mL) and AL (25 mL) milling jars were manufactured in house to match the internal dimensions of the Retsch models using chemical resistant slippery PTFE and multipurpose 6061 aluminum purchased from McMaster Carr; associated milling balls (12.7 mm) of identical material were purchased from McMaster Carr. Two types of in-house AL jars were utilized, one jar type sealed shut with traditional threads which broke halfway through trials due to thread warping upon prolonged use causing issues preventing jar opening and sealing. To replace this model and ensure the same issue did not recur, we manufactured jars capable of sealing with a KF toggle clamp matching our previously reported hermetically sealed jar design described in Figures S1 and S2.[1]

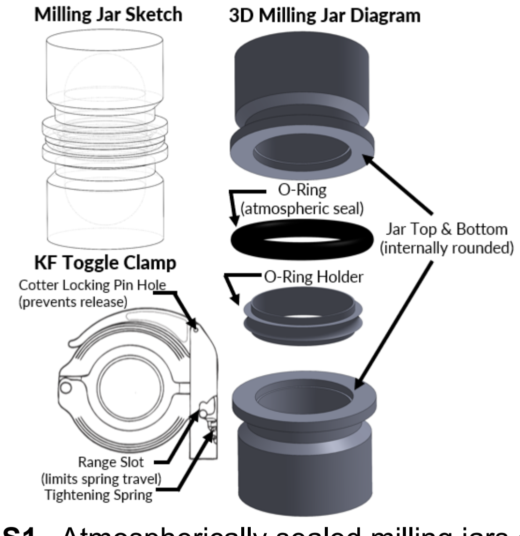

Figure S1. Atmospherically sealed milling jars diagram.

The milling jar cavity consists of two hemispheres (diameter = 1.02 inches, radius of curvature = 0.51 in) connected by the middle of the o-ring (diameter = 1.02 inches, height = 0.153 inches) and a cylinder (diameter = 1.02 inches, height = 2 x 0.49 inches = 0.98 inches).

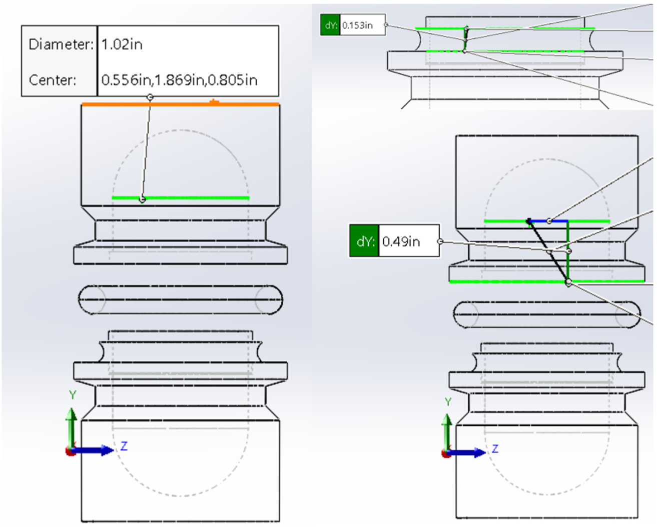

Figure S2. SolidWorks images showing relevant milling jar internal cavity measurements.

Pictures of all milling jar systems used in this study are provided in Figure S3.

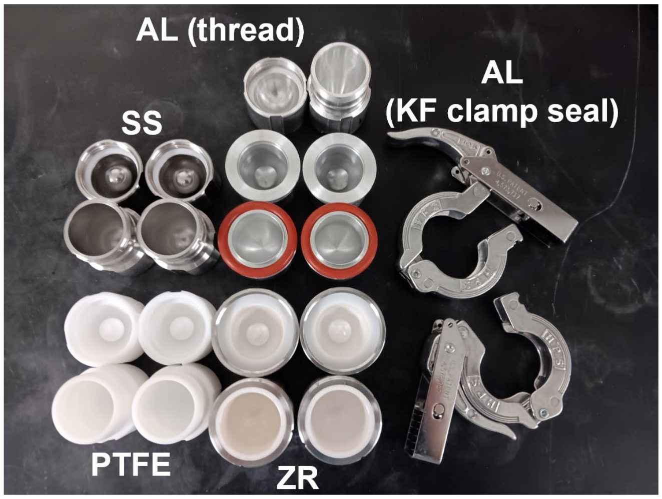

Figure S3. Pictures of all the milling jars of various materials utilized in this study.

2.1.2 Milling Ball Sizes and Weights

Milling balls utilized for the experiments were as follows:

- SS 15 mm diameter, 13.40 g
- SS 12.7 mm diameter, 8.55 g
- AL 12.7 mm diameter, 2.99 g
- PTFE 12.7 mm diameter, 2.30 g
- ZR 12.7 mm diameter, 6.52 or 5.49 g (these balls differed by more than typical between different balls of within +/- 0.05 g of one another, but this difference was insufficient to effect noticeable change in reactivity and thus, trials are a mixture of both ball sizes)

2.1.3 System for Putting Different Materials in Different Milling Jar Regions

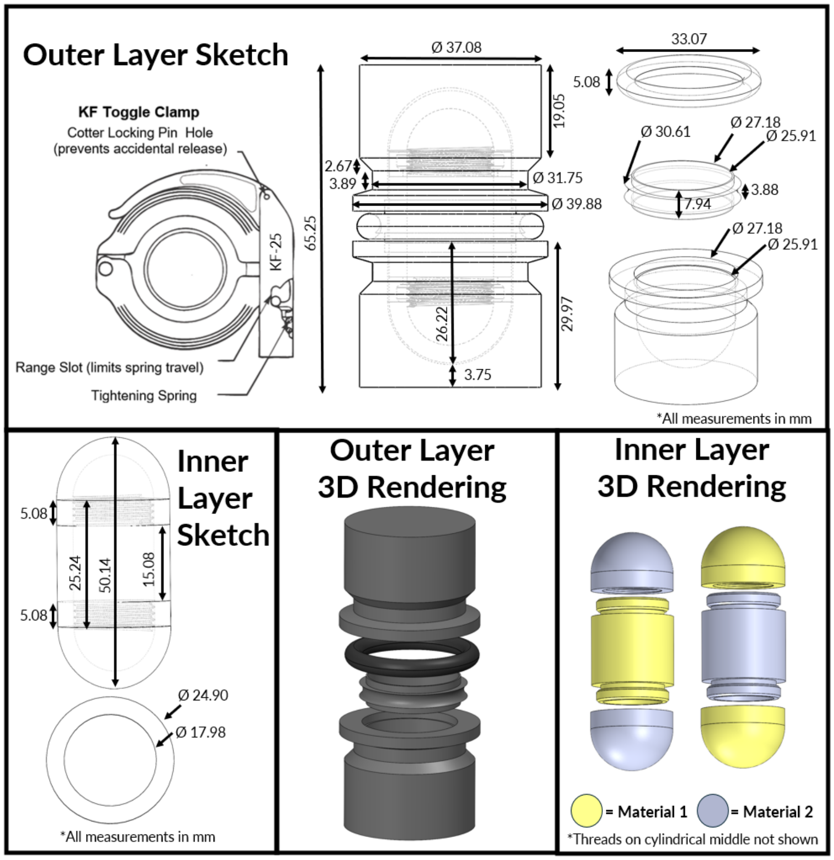

Figure S4. Sketch of the prototype system for putting different materials under different atmospheres in different jar regions with associated measurements and 3D CAD rendering of the device.[2]

The milling material for the jar inserts was 316 stainless steel and chemical resistant slippery PTFE purchased from McMaster Carr.

2.2 NMR

2.2.1 Instrumentation

^{1}HNMR spectra were obtained with either 1) a Bruker Advance Neo 400 instrument equipped with a 400 MHz Ascend magnet, an automated tuning 5 mm broadband iProbe, and a 60 position SampleXpress sample changer or 2) a 500 MHz Varian system equipped using a Varian VnmrS console equipped with an Oxford magnet, and 5 mm 1H [X] broadband and [1H/19F] [X] switchable probes.

2.2.2 NMR Reference Data

NMR reference data for the condensation product matches literature reports (within +/- 0.03 ppm)[3]. Product peaks are as follows:

5-(4-hydroxy-3-methoxybenzylidene)pyrimidine-2,4,6(1H,3H,5H)-trione – Orange powder, m.p. 263 °C.[3] **^{1}H NMR (400 MHz, d_6-DMSO):** 2.50 (DMSO), 3.33 (H_2O), 3.83 (3 H, s, CH_3), δ 6.89

(1 H, d, J = 8.38, Ar), δ 7.81 (1 H, dd, J = 1.69, 8.48, Ar), δ 8.23 (1 H, s, OH), δ 8.48 (1 H, d, J = 1.68, Ar), δ 10.55 (1 H, s, CH), δ 11.14 (1 H, s, amine), δ 11.26 (1 H, s, amine).

2.3 PXRD

The sample was placed in the sample holder of a two circle goniometer, enclosed in a radiation safety enclosure. The X-ray source was a 1kW Cu X-ray tube, maintained at an operating current of 40 kV and 25 mA. The X-ray optics was the standard Bragg-Brentano para-focusing mode with the X-ray diverging from a DS slit (1mm) at the tube to strike the sample and then converging at a position sensitive X-ray Detector (Lynx-Eye, Bruker-AXS). The two-circle 218 mm diameter θ-θ goniometer was computer controlled with independent stepper motors and optical encoders for the θ circle with the smallest angular step size of 0.0001 to 2θ. The software suit for data collection and evaluation is windows based. Data collection is automated COMMANDER program by employing a DQL file. Data is analyzed by the program EVA.

Experimental Parameters

- Parent: 2Theta Coupled
- Scan Type: TwoTheta/Theta
- Scan Mode: Continuousscan
- Scan Status: Completed
- Start: 5.0001
- End: 40.00067
- Step Size: 0.01964117
- Goniometer Radius: 250
- Theta: 2.50005
- Anode: Cu
- Focus Orientation
    - kα1: 1.5406
    - kα2: 1.54439
    - kα2: Ratio 0.5
    - kβ: 1.39222
- Kβ Filter Wavelength for display: 1.5406
- Generator kV: 40
- Generator mA: 25
- Detector Name LynxEye
- LynxEye 0D: No
- Detector Opening: 2.977188
- Air-Scatter Screen: Yes
- Slit Mode: Fixed
- Simul. Slit Mode
    - Creation Date/Time: 10/25/2024 10:24
    - Last Write Date/Time: 10/25/2024 10:24
- Measurement Duration: 7.03:04:19

2.4 Temperature measurements

The temperature of the milling jar for different systems at different time points during milling were measured using an IR thermal camera (FLIR TG165-X) and a K-type thermocouple (-40 – 1000°C) attached to a digital multimeter (Extech Instruments EX355). The 25 mL jars and inserts

were loaded with reactants as described in Materials and Methods section 2.4.1 and 2.5. For IR thermal images, a laser was used as a guide for taking spot temperature measurements. Thermal images were recorded with the device emissivity settings at 0.95. All IR images were taken within one minute of the mill stopping. Meanwhile, thermocouple measurements were taken within two minutes of the mill stopping.

2.5 Autocatalysis experiments

To simulate the reaction at 20% conversion, the amount of the reactants and the product needed to get the same total mass (0.5325 g) with mole ratios of 8 mmol vanillin: 8 mmol barbituric acid: 2 mmol product were calculated. For these experiments no further purification of the crude product mixture was done, and so the amount of water present in the product (17 wt%) was accounted for when the reactant masses were calculated. For the reactions seeded with product, vanillin (1.48 mmol), barbituric acid (1.48 mmol), product (0.37 mmol), and 10 wt% water were added to a 25 mL jar (SS or PTFE) along with a 12.7 mm ball. This jar was loaded onto one side of the Retsch MM400 mill. Meanwhile, a jar containing the reaction system without the product with amounts (see SI section 3) was loaded onto the other side of the mill. These two reactions were run at 30 Hz for specified times, and the conversions were measured using ^{1}H-NMR as described in SI section 2.2. A total of three measurements for each time period was collected. To account for the initial amount of product added, the % conversion was calculated using the formula below, where A_R is the area integration of the vanillin peak at 9.76 ppm and A_P is the area of integration of the product peak at 6.90 ppm.

$$\% \, Conversion = \left(1 - \frac{1.25\, A_R}{A_R + A_P}\right) \times 100\, \%$$

3. GENERAL PROCEDURES FOR 5-(4-HYDROXY-3-METHOXYBENZYLIDENE)PYRIMIDINE-2,4,6(1H,3H,5H)-TRIONE SYNTHESIS

3.1 Experiments Tracking the Kinetics in Different Materials

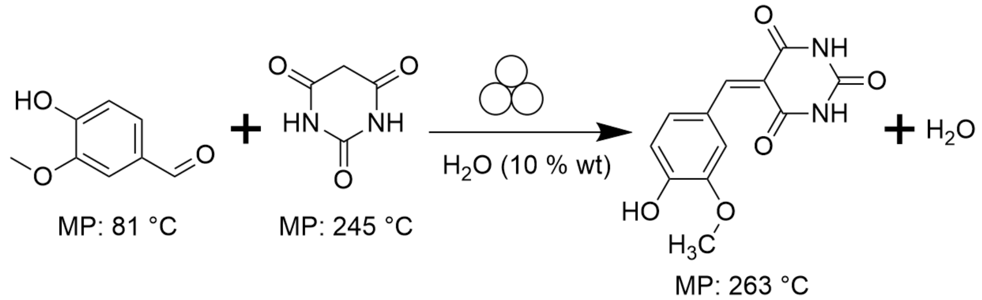

Figure S5. Reaction synthesis scheme.

Chemicals were purchased from BeanTown Chemical and used without further purification. Synthesis of 5-(4-hydroxy-3-methoxybenzylidene)pyrimidine-2,4,6(1H,3H,5H)-trione was performed in a Retsch MM400 mixer mill operating at a frequency of 30 Hz or 25 Hz using the materials described in section 2.1.

In a typical experiment, milling media were first cleaned with acetone, water, and Alconox soap followed by a rinse with DI water and drying. Then, the milling ball (see section 2.1.2), vanillin (1.9 mmol), barbituric acid (1.9 mmol), and water (2.9 mmol, 10 % wt) were loaded into a 25 mL

milling jar (see section 2.1.1). Jars were then placed on a Retsch MM400 mill and milling was performed for the desired run time at either 25 Hz or 30 Hz. Upon reaction completion, a small amount of crude product (typically 5-25 mg) was collected from multiple jar regions and immediately dissolved in DMSO-d_6 (between 3-7 minutes following milling end, 750-800 μL). Conversion was measured by ^{1}H nuclear magnetic resonance spectroscopy (NMR) (see SI section 2.2).

3.2 A Note on Reaction Progression After Milling in the Solid

Due to previous reports that these reactions can proceed in the solid-state following milling,[3, 4] we dissolved materials in DMSO-d_6 directly after milling was ended and proceeded to take NMR measurements. All experiments were done as single point trials to the desired measurement time to avoid potential pauses in milling thus ensuring kinetic data was reflective solely of the mechanochemical process and not reflective of further reaction after milling paused or other cooling effects due to pauses in the grinding process.

3.3 A Note on Reaction Progression in Solution

The reaction can proceed in solution, but the rate is very slow as described in the literature.[3] Given this fact along with the comparatively low concentrations utilized herein (5-25 mg in 750-800 μL) compared to literature concentrations (70 mg in 600 μL) for solution kinetics tracking; we suspected this would not preclude getting representative % conversion measurements by NMR as long as solution was not left in tube for an extended period of time before measurement. As a control, initial test reactions at only 38.0% and 35.8% conversion by NMR (after 10 and 20 minutes of being in solution respectively) were left in the NMR tubes for 1 hr and 40 minutes and showed no measurable change in yield. After a further 2 days and 22 hrs, NMR was again performed to measure conversion. In the first sample, reaction mixture conversion rose by ~2% to 40.3% while in the second sample conversion had risen by ~5% to 41.1%. These values are within the error of most NMR conversion measurements ensuring representative measurements of % conversion are attainable. As an extra precaution, we sought to get NMR measurements within 12 hours of dissolution, but often were able to go even faster to within 1 hour or less. Experiments with longer times in solution were also checked against replicates with shorter solution times to ensure no significantly large conversion differences were observed which would suggest more reactivity in solution than expected.

4. DETERMINING THE ONSET OF THE FEEDBACK PERIOD: 1ST and 2ND DERIVATIVES OF REACTION KINETICS FITS

Derivatives are taken from the kinetic fits of the reaction derived from the model presented in the article. The program Igor Pro 9 was utilized to determine derivatives using the central differences algorithm employing approximate endpoints. For second derivatives, binomial smoothing was applied as needed. Smoothing values are given on derivative plots for each curve, where a value was not shown, no smoothing was necessary to clearly define the maximum position.

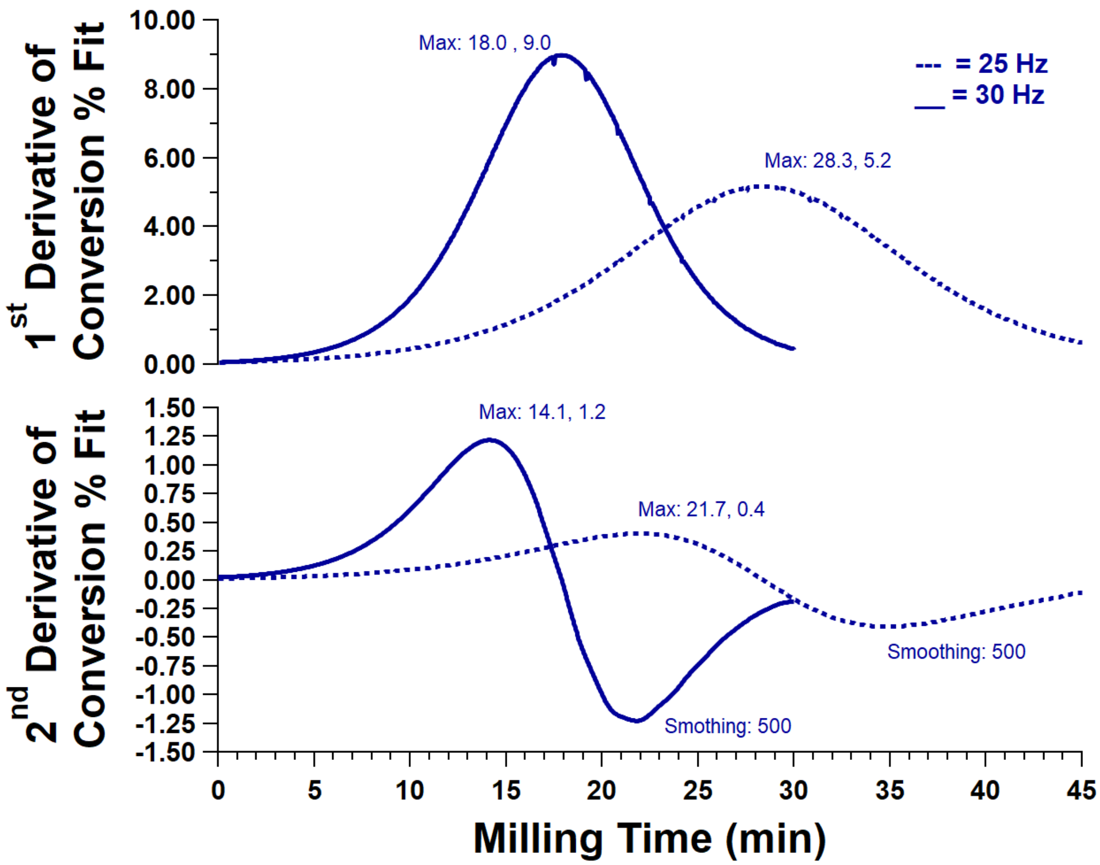

Figure S6. Derivatives of the kinetic fit of the reaction performed with a 15 mm SS ball at various frequencies used to determine the onset of the feedback period discussed in the text.

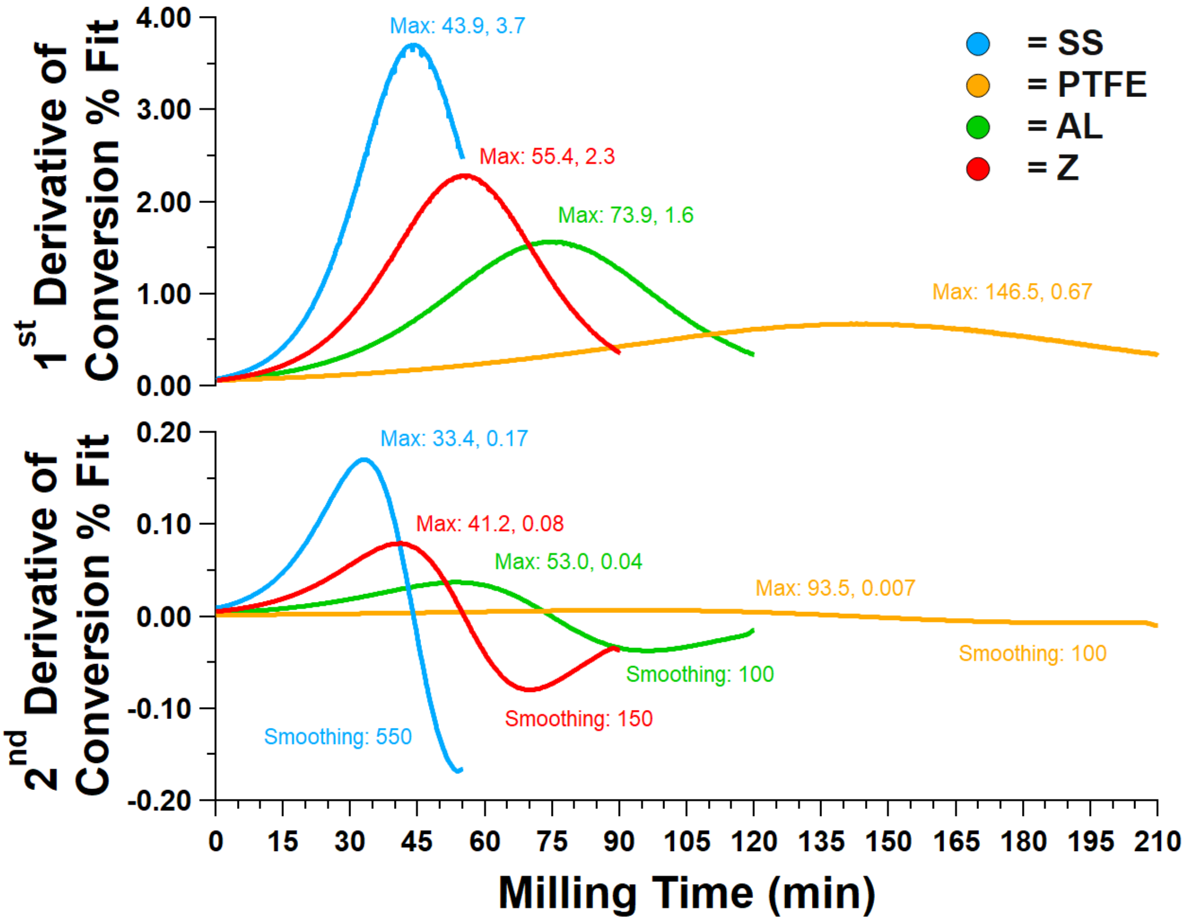

Figure S7. Derivatives of the kinetic fit of the reaction performed with a 12.7 mm ball at 25 Hz in a variety of materials used to determine the onset of the feedback period.

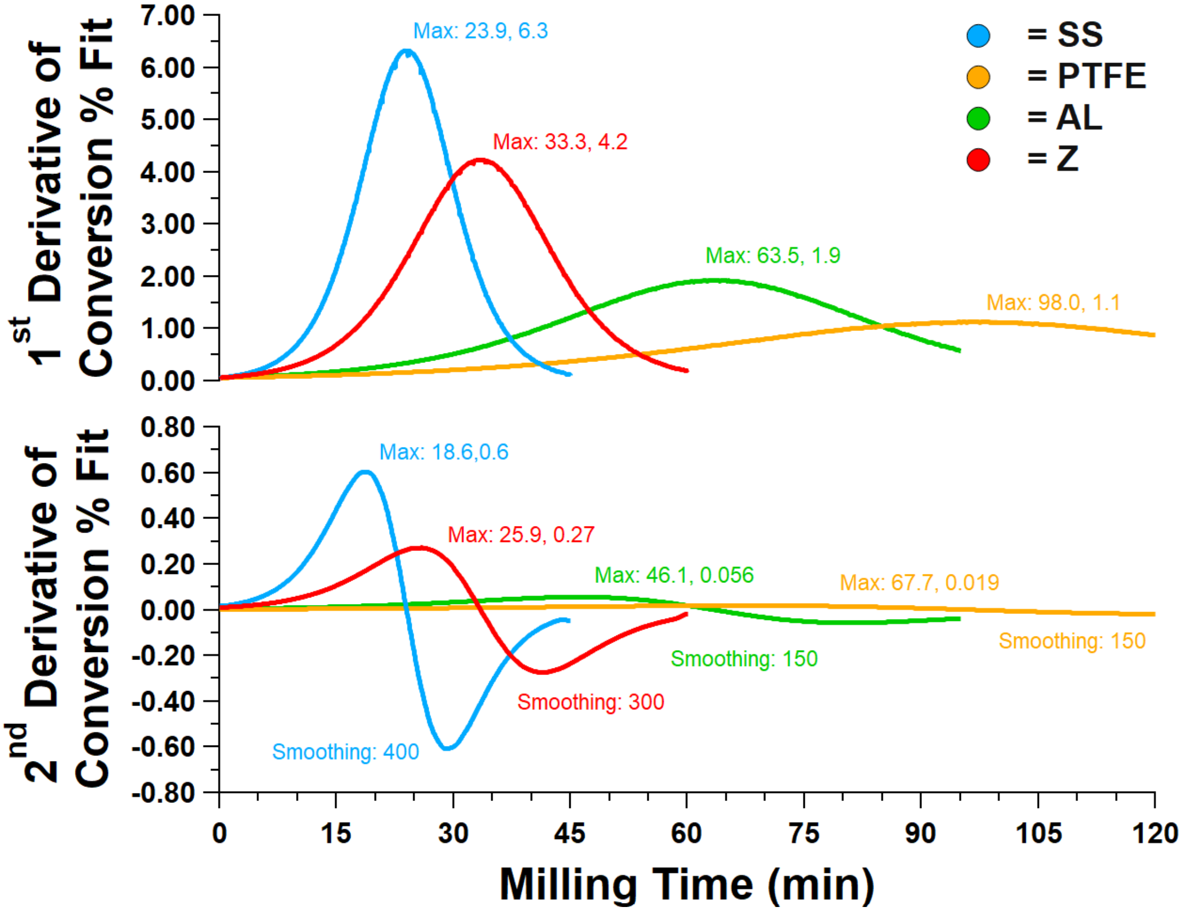

Figure S8. Derivatives of the kinetic fit of the reaction performed with a 12.7 mm ball at 30 Hz in a variety of materials used to determine the onset of the feedback period.

5. RHEOLOGY IN STAINLESS STEEL AS A FUNCTION OF FREQUENCY AND BALL SIZE

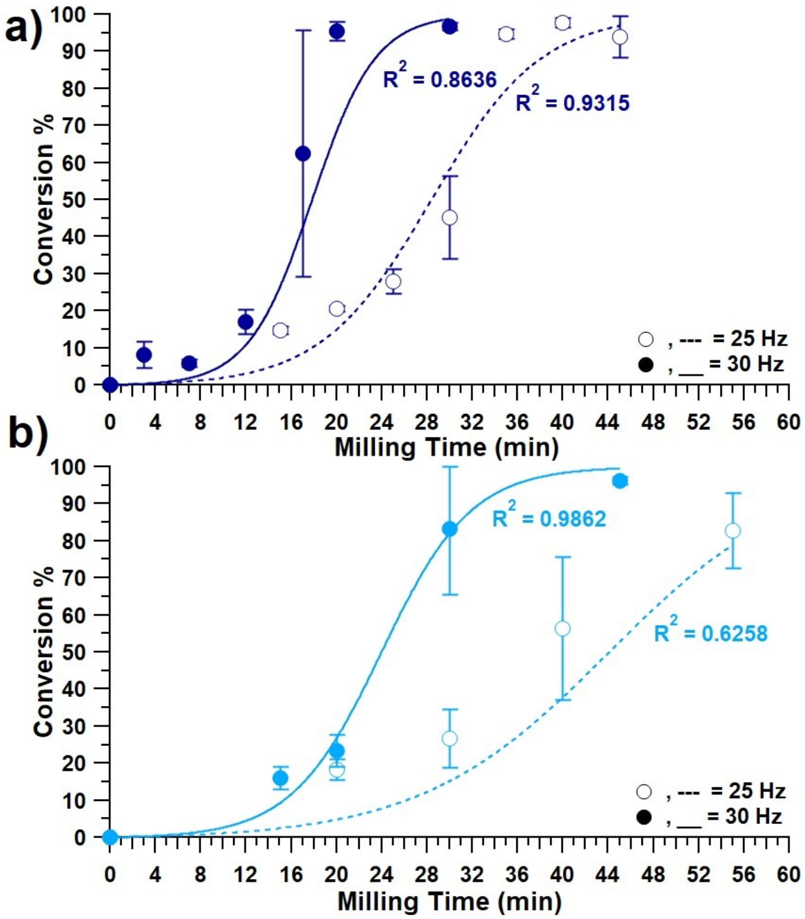

Figure S9. Reaction kinetics in SS at 25 and 30 Hz using a) a 15 mm SS ball and b) a 12.7 mm SS ball. All curves shown were replicated by four trials and error is given as standard deviation. Curves fit using autocatalytic model described in text.

The reaction exhibits sigmoidal kinetics with a feedback period initiating at 21.7 minutes into the milling at 25 Hz once 20.1 % conversion was reached as shown by Figure S6a. Upon increasing the frequency to 30 Hz, the feedback period begins similarly around 20.7 % conversion which is reached at 14.1 minutes into milling. This is in line with previous reports.[3] The formation of the snowball is more difficult to replicate. As reported previously, the snowball can be found roughly in the center of the feed-back period.[3] However, this only occurred in half of the trials. In other trials, the powder appeared to preferentially adhere to, and form a coating on, the vessel walls as shown in Figure S7. This occurred at both 25 and 30 Hz in the corresponding feedback periods. When removed from the walls or milling ball, the material appears plastic or glass-like and requires scraping to be broken into brittle, large, stable chunks. The general curved solid shapes impressed in the material by adherence to the vessel or ball remained, and we did not observe the material reverting to a powder-like state with the sample remaining stable for up to 2 weeks, contrary to literature reports.[3]

Upon decreasing the size of the milling ball to 12.7 mm, the feedback period at 30 Hz was observed to begin at 18.6 min and 20.4 % conversion with an associated slower kinetic acceleration within the sigmoidal curve as shown by the visual “flattening” of the curve in Figure S6b. At 25 Hz the feedback period is similarly delayed to 33.4 minutes at 21.5 % conversion and “flattened”. Thus, it can be generally noted that feedback begins when the mixture reaches about 20 % conversion. The time until this value is reached and positive feedback begins varies as a function of frequency and ball size. This explains why others have not observed a similar feedback period at 15 Hz as the kinetics of the reaction were only tracked to 20% conversion under identical conditions. As in the case of the 15 mm ball, the snowball—or a glassy coating on the outside of the vessel—was observed during the feedback period at 30 Hz. However, no snowball was observed with the smaller ball at 25 Hz.

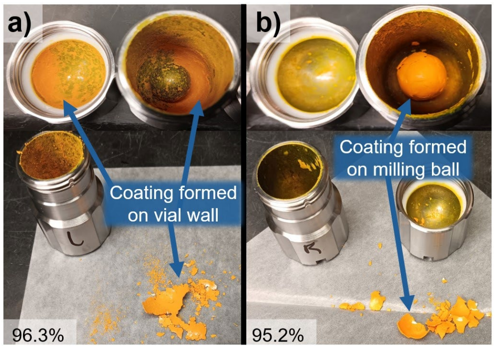

Figure S10. Material coating jars after 17 minutes of milling at 30 Hz with a 15 mm diameter SS ball (13.4 g) showing a) the formation of the coating on the vessel walls and b) the coating on the milling ball (aka. snowball). The conversion % reached in each appearance is provided. It should also be noted that these trials were run simultaneously each on one side of the mill.

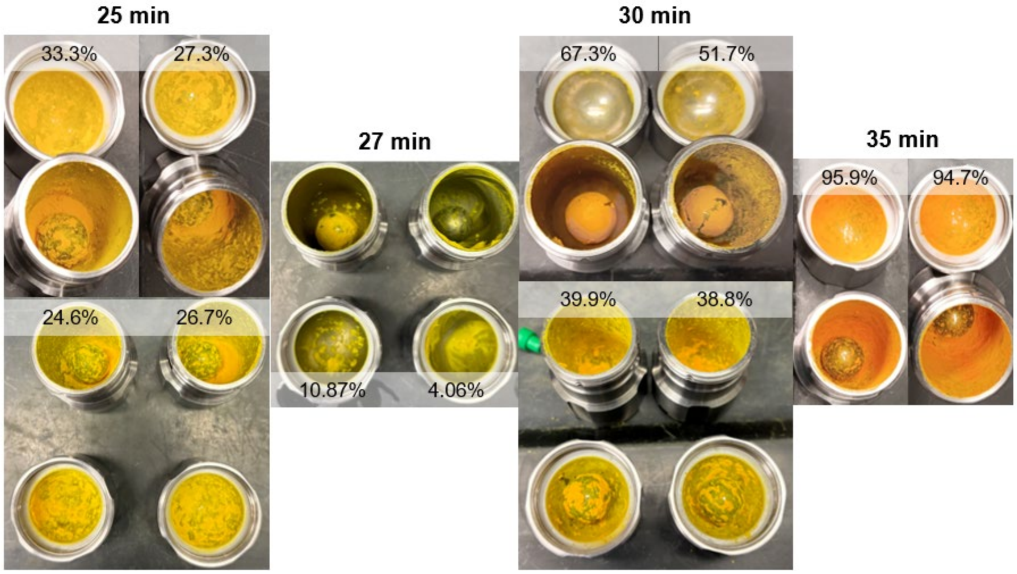

Figure S11. Reaction mixture rheology at different time points in SS (25 mL) jar with a 15 mm ball operating at 25 Hz.

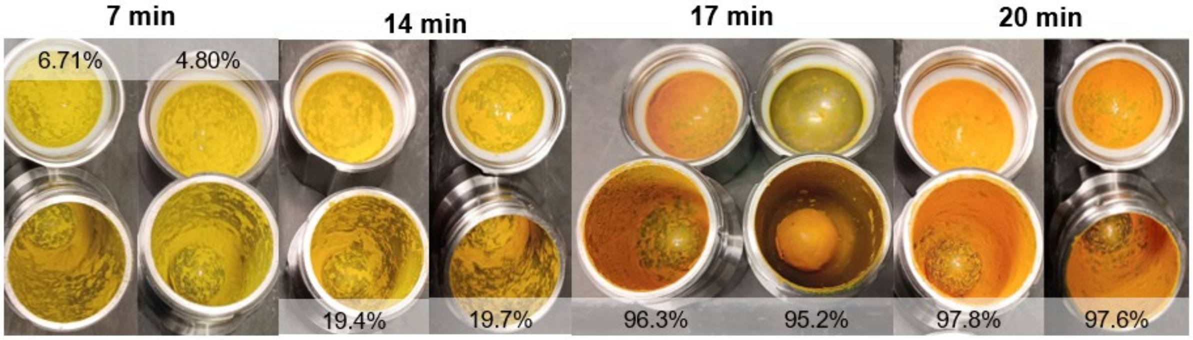

Figure S12. Reaction mixture rheology at different time points in SS (25 mL) jar with a 15 mm ball operating at 30 Hz.

6. FITTING THE KINETICS TO TRADITIONAL MODELS

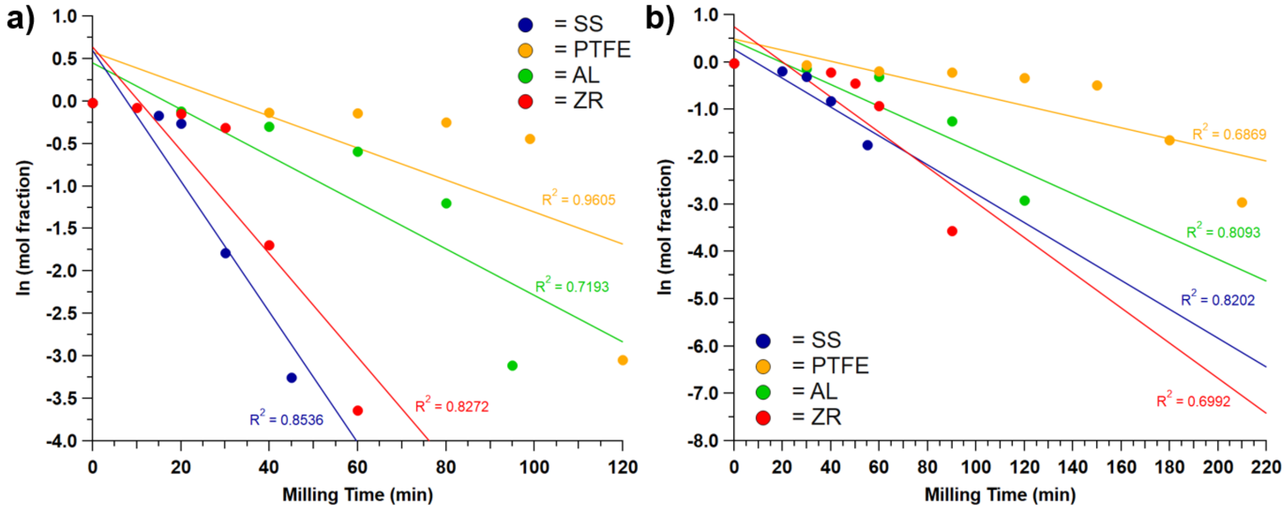

Figure S13. Attempt at first order kinetics fits of each material with 12.7 mm balls at a) 30 Hz and b) 25 Hz.

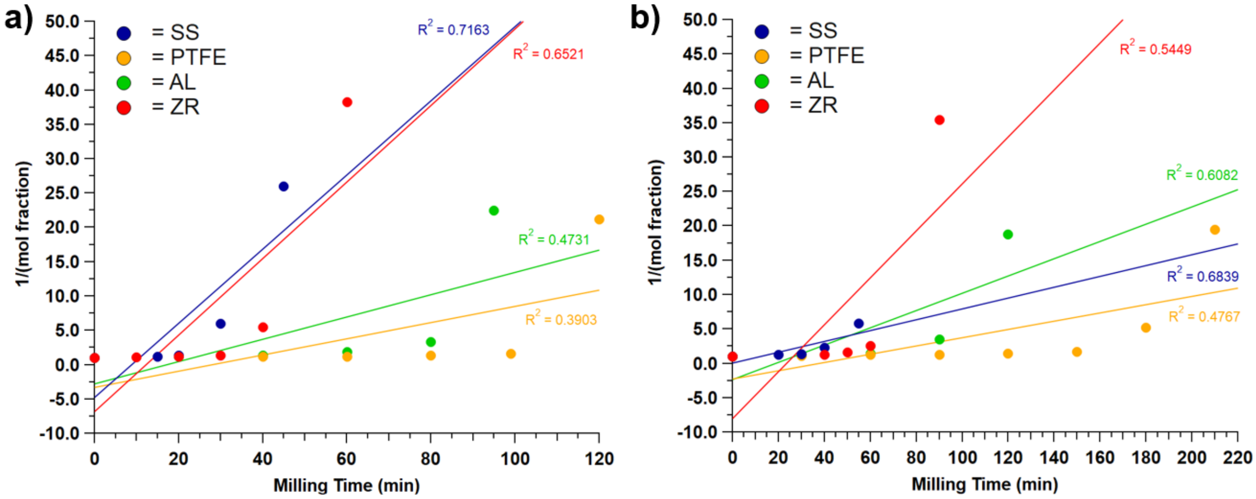

Figure S14. Attempt at second order kinetics fits of each material with 12.7 mm balls at a) 30 Hz and b) 25 Hz.

7. IMPACT FORCE MODEL IN A VIBRATORY BALL MILL

Consider a reciprocating ball mill that translates a distance L with a frequency of f. If we further assume that, on average, the ball within the mill has a speed of zero relative to the global frame of reference, the ball speed is given with respect to the reciprocating mill as,

$$v = 2fL \tag{1}$$

The ball then impacts the vessel's side with an energy $K = 0.5mv^2$ where m is the ball's mass. Some fraction of the energy φ transfers from the ball to the vessel wall. Under the assumption of elastic collisions such as linear elasticity, neglecting shear stresses, small deformations and no adhesion between contacting surfaces, the impact force can be related to the transfer of energy using Hertz contact mechanics between a sphere and a half space,

$$K = Fd \tag{2}$$

$$K = Fd = \frac{1}{2}mv^2 \tag{3}$$

where d is the indentation depth. Using the elastic modulus, E and Poisson ratio, v for the ball and vessel materials, effective mechanical modulus $E^*$is given by,

$$E^* = \left[\frac{1-v_1}{E_1} + \frac{1-v_2}{E_2}\right]^{-1} \tag{4}$$

The radius of impact is expressed in terms of the radius of ball R_b and radius of reaction vessel curved ends R_c.

$$R = \left(\frac{1}{R_b} - \frac{1}{R_c}\right)^{-1} \tag{5}$$

Expressing kinetic energy of the ball in terms of ball parameters gives,

$$F = \frac{1}{2}\left(\frac{4}{3}\pi\rho R_b^3\right)v^2 = \frac{2}{3}\pi\rho R_b^3 v^2 \tag{6}$$

ρ is the ball density. Next, we solve for d using equation

$$d = \frac{0.5mv^2}{F} = \frac{\frac{1}{2}\left(\frac{4}{3}\pi\rho R_b^3\right)v^2}{F} \tag{7}$$

By substituting d in to the expression for F from Hertz contact framework:

$$F = \left(\frac{4}{3}\right)E^* R^{\frac{1}{2}}\, d^{\frac{3}{2}} = \left(\frac{4}{3}\right)E^* R^{\frac{1}{2}}\left(\frac{\frac{1}{2}\left(\frac{4}{3}\pi\rho R_b^3\right)v^2}{F}\right)^{\frac{3}{2}} \tag{8}$$

Combining all terms and expressing in terms of F,

$$F = \left(\frac{4}{3}\right)E^* \frac{2^{3/2}}{3} R^{1/2}\left(\rho\cdot\pi\cdot R_b^3\cdot v^2\right)^{\frac{3}{2}} F^{-\frac{3}{2}} \tag{9}$$

$$F^{\frac{5}{2}} = \left(\frac{4}{3}\right) E^* \left(\left(\frac{1}{R_b} - \frac{1}{R_c}\right)^{-1}\right)^{1/2} \frac{2^{3/2}}{3} \left(\rho \cdot \pi \cdot R_b^3 \cdot v^2\right)^{\frac{3}{2}} \tag{10}$$

Rearranging and grouping constants and expressing force in terms of frequency, length of milling media travel, geometric, and material properties gives,

$$F = 1.75 \left[E^* \left(\left(\frac{1}{R_b} - \frac{1}{R_c}\right)^{-1}\right)^{\frac{1}{2}} \left(\rho \cdot \pi \cdot R_b^3 \cdot (2 \cdot l \cdot f)^2\right)^{\frac{3}{2}}\right]^{2/5} \tag{11}$$

Equation 11 shows that the force depends on the density of the milling media, collision frequency, length of ball travel, and material.

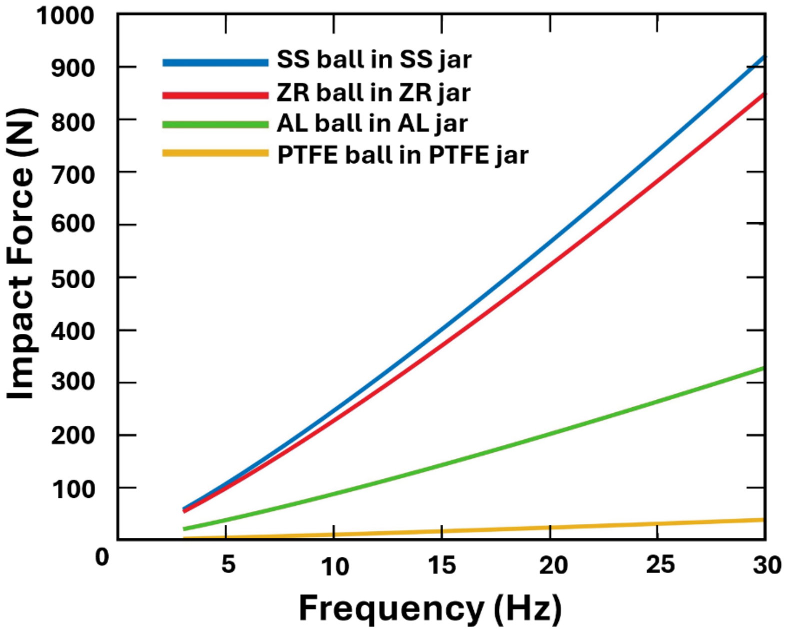

Figure S15. Computed impact forces as a function of milling frequency and reactor mechanics

8. EXPERIMENTS WITH DIFFUSION DRIVEN REACTIVITY

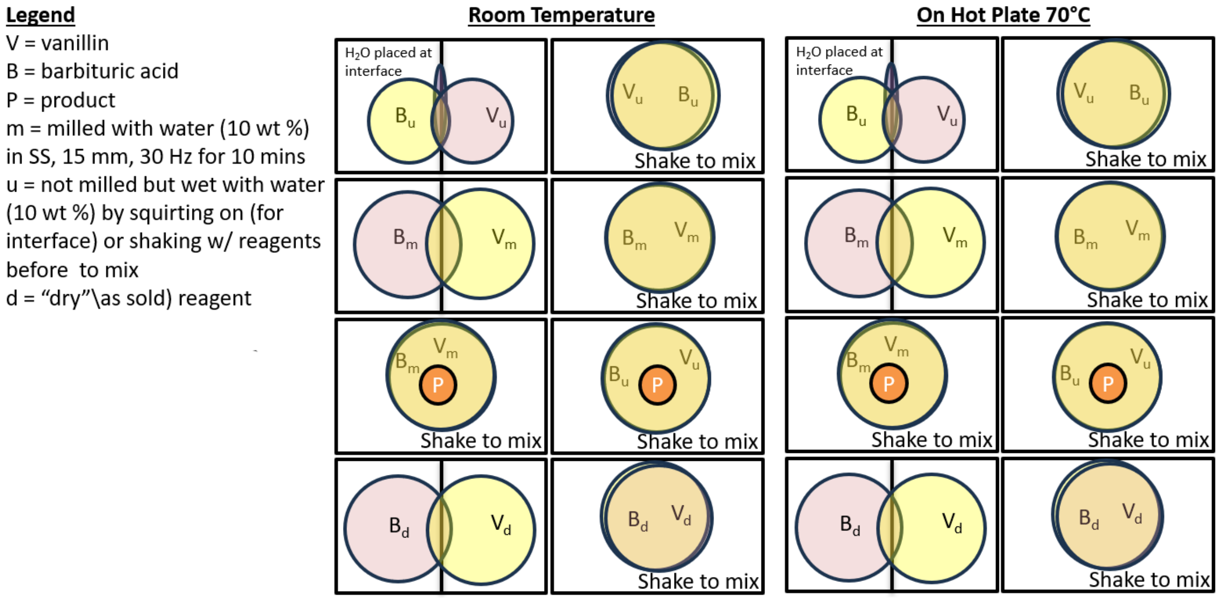

Figure S16. Summary of experiments looking at continuing reaction between reagent powders with different pre-treatments.

Knoevenagel condensations have been reported to occur in the solid state via direct heating.[4] We have similarly observed reaction progress after milling is ended in the case of vanillin and barbituric acid. To further explore this effect, we have performed a series of experiments looking at reaction along interfaces between powders at different temperatures in hope of further elucidating effects that could play a role in the feedback period observed in the mechanochemical reaction. A summary of these experiments is shown in Figure S16. Reaction procedures and results are as follows:

8.1 Interactions Between Dry Reagents

Reagents directly from suppliers without additional water addition were utilized as controls. Upon placing the reagents in contact, yellow color slowly forms at the interface and slowly diffuses outward from the vanillin into the barbituric acid as shown in Figure S17. Similar behavior is shown when heating the mixture, but the rate is slightly accelerated. Pre-shaking of the reagents together also results in slow yellow color formation at both temperatures observed (Figure S17).

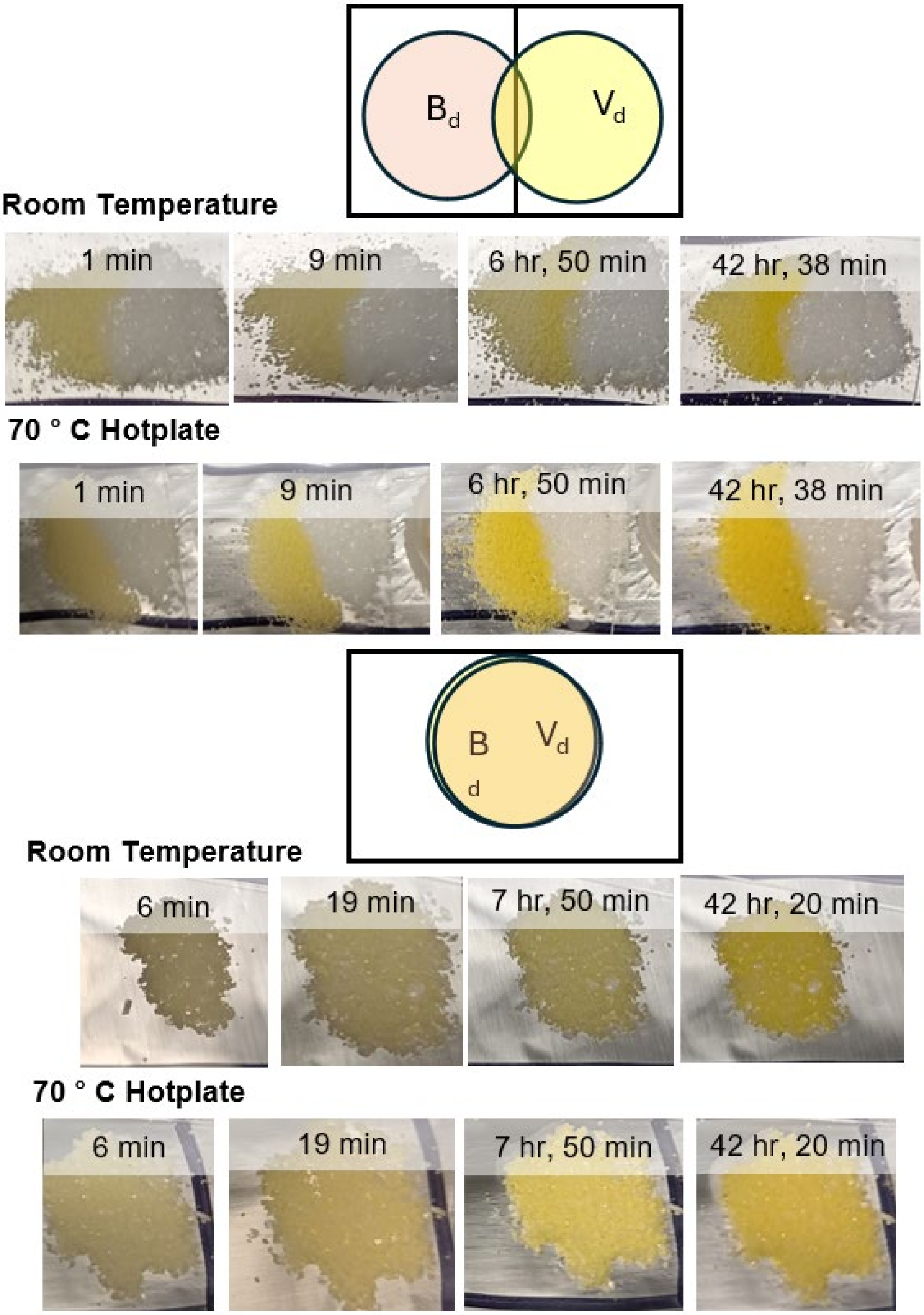

Figure S17. Mixtures of milled reagents pressed together to make an interface (top) or shaken together and left (bottom) at both room temperature and 70 °C.

8.2 Interactions Between Pre-Milled Wet Reagents

Reagents were separately pre-milled with water (10 % wt) in a SS (25 mL) Retsch MM 400 vessel using a SS milling ball (15 mm, 13.4 g). This results in white/off-white powders (monitoring up to 1 week was performed with no observed changes in the powder coloration or appearance). Subsequently, reagents were pressed together and observed at both room temperature and on a hot plate set to 70 °C. At room temperature, the products begin as white/ off white powders. Upon touching yellow color forms at the interface and slowly diffuses outward from the vanillin into the barbituric acid as shown in Figure S17. In the case of 70 °C heating, formation of yellow color is

faster and followed rapidly by orange product formation at the interface as shown in Figure S15. Taking the milled reagents and shaking them together then leaving them together shows similar behavior to that seen at the interface with slow yellow color formation appearing at room temperature with the formation of yellow product quickly followed by orange at 70 °C heating (Figure S18).

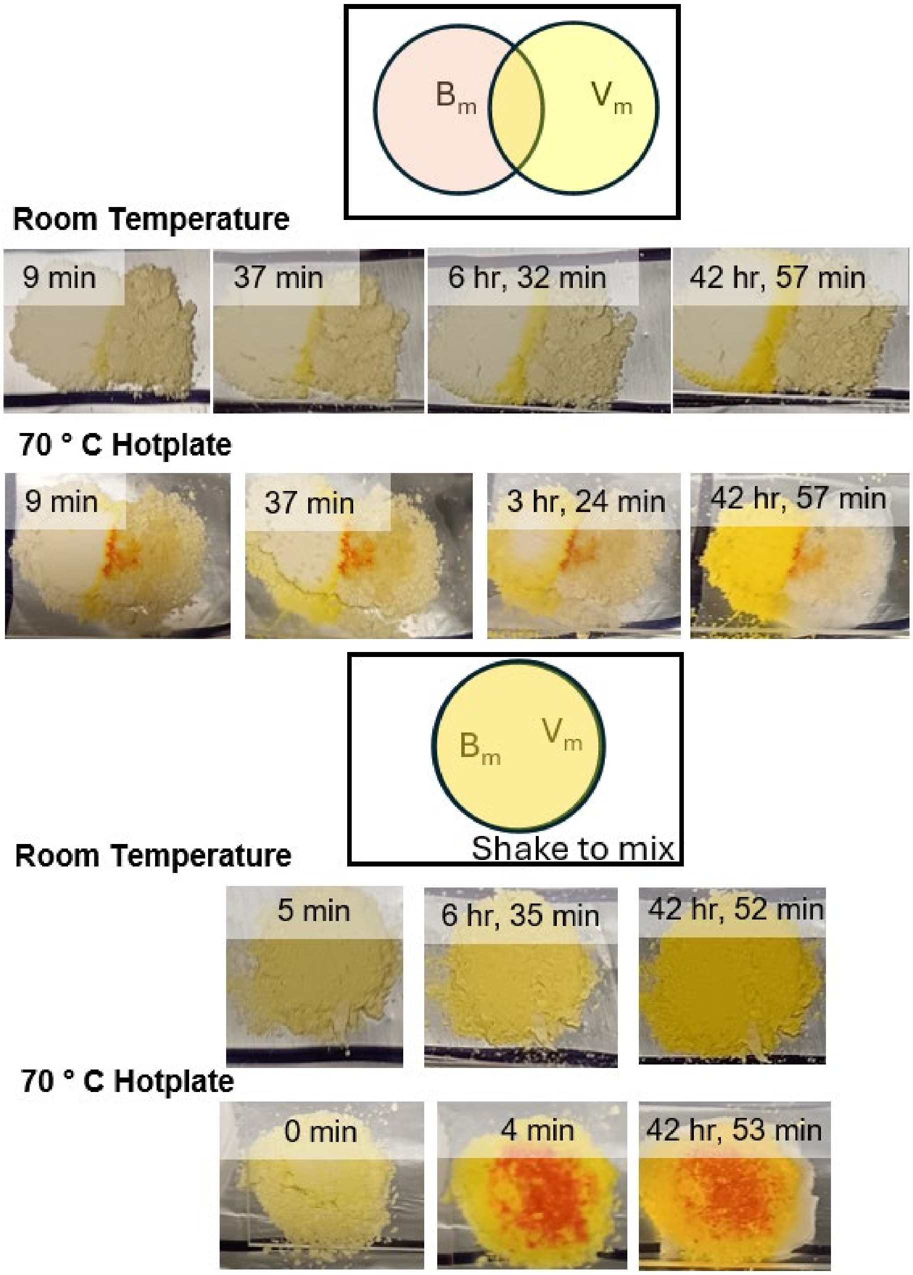

Figure S18. Mixtures of milled reagents pressed together to make an interface (top) or shaken together and left (bottom) at both room temperature and 70 °C.

8.3 Interactions Between Wet Reagents (Without Pre-Milling)

To explore the reaction at the interface, dry reagents were placed together and subsequently 21 μL of water was injected at the interface. In the case of the room temperature trials, yellow

coloration quickly followed water addition within the barbituric acid followed slowly by orange product formation emanating from the vanillin through the barbituric acid as shown in Figure S18. At 70°C, water addition at interface spreads yellow color through the barbituric acid till water is spent quickly followed by orange product formation in regions of yellow coloration which seems to largely halt and be subsequently followed by yellow coloration slowly spreading through the rest of the matrix in regions where water didn't travel (Figure S18).

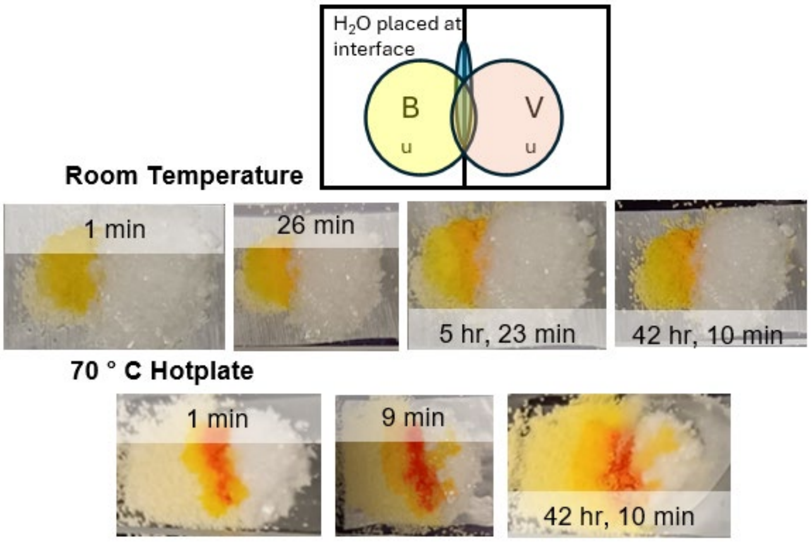

Figure S19. Mixtures of dry reagents pressed together to make an interface and subsequently squirted with 21 μL of water at the interface.

To explore the effect of additional water without pre-milling, reactants were shaken together (1:1 mol ratio) with water (10 wt %) and left to sit at both room temperature and 70°C. Reactants quickly seem to take on a bright yellow color when shaken together in the presence of water. Subsequently, the reaction mixture turns orange which happens more rapidly at higher temperatures as shown in Figure S20.

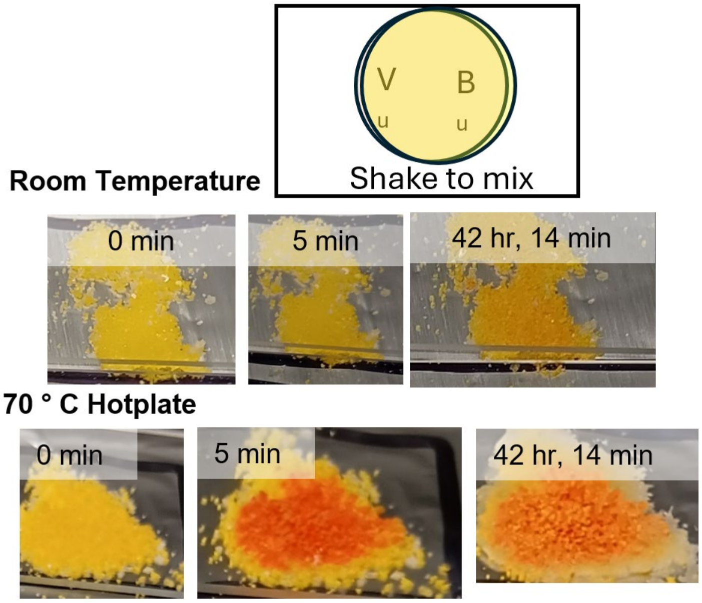

Figure S20. Mixtures of wet reagents (without pre-milling).

8.4 Experiments Testing Product Autocatalysis

To test for potential autocatalytic effects, reagents were separately pre-milled with water (10 % wt) in a SS (25 mL) Retsch MM 400 vessel using a SS milling ball (15 mm, 13.4 g) then shaken together in a 1:1 mol ratio. Subsequently pre-synthesized product was placed within the reagent mixture and monitored. At room temperature yellow color forms through the mixture quickly at a rate which tentatively appears faster than that of milled reagents forming yellow coloration without existing orange product as shown in Figure S20. However, the formation of yellow color does not seem to emanate from the pre-existing orange powder so clear conclusions cannot be drawn. At 70 °C, yellow coloration does seem to emanate more directly from around the existing orange product and subsequently leads to fast formation of further yellow coloration and subsequently orange product which emanates out from the existing product as shown in Figure S20. However, the process at high temperature ceases after initial rapid product formation and fails to continue to diffuse significantly throughout the matrix despite further yellow coloration.

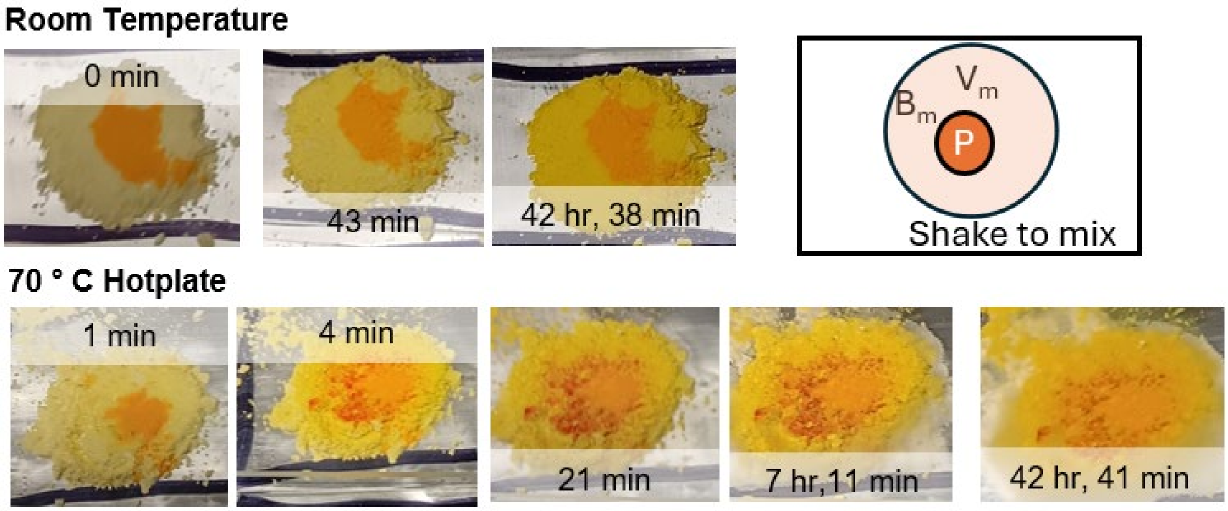

Figure S21. Experiments testing autocatalysis with pre-milled reagents.

For further tests, reactants were shaken together (1:1 mol ratio) with water (10 wt %) and left surrounding pre-synthesized product to sit at both room temperature and 70°C. As with controls using pre-milled reagents at room temperature, yellow coloration is already formed by shaking the reagents in the presence of water. Subsequently, the mixture turns an orange color much faster than in the previous case without the presence of existing product (which only shows slight orange product formation after 42 hours as opposed to the near total orange coloration observed herein at 30 min) as shown in Figure S21. However, it should be noted that orange coloration could not be clearly observed to emanate from existing product. In the case of 70 °C, orange product formation happens rapidly and as in the milled case, occurs near existing product while similarly failing to propagate outward after initial reaction as shown in Figure S22.

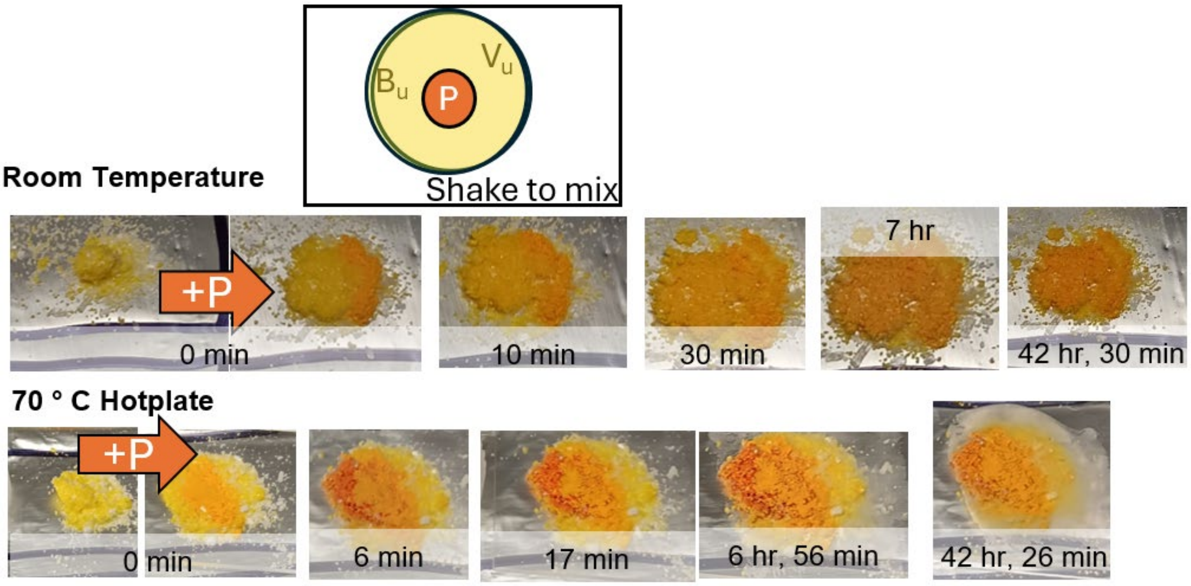

Figure S22. Experiments testing autocatalysis with wet reagents not subjected to milling treatment.

8.5 PXRD to Identify the Yellow-Colored Compound

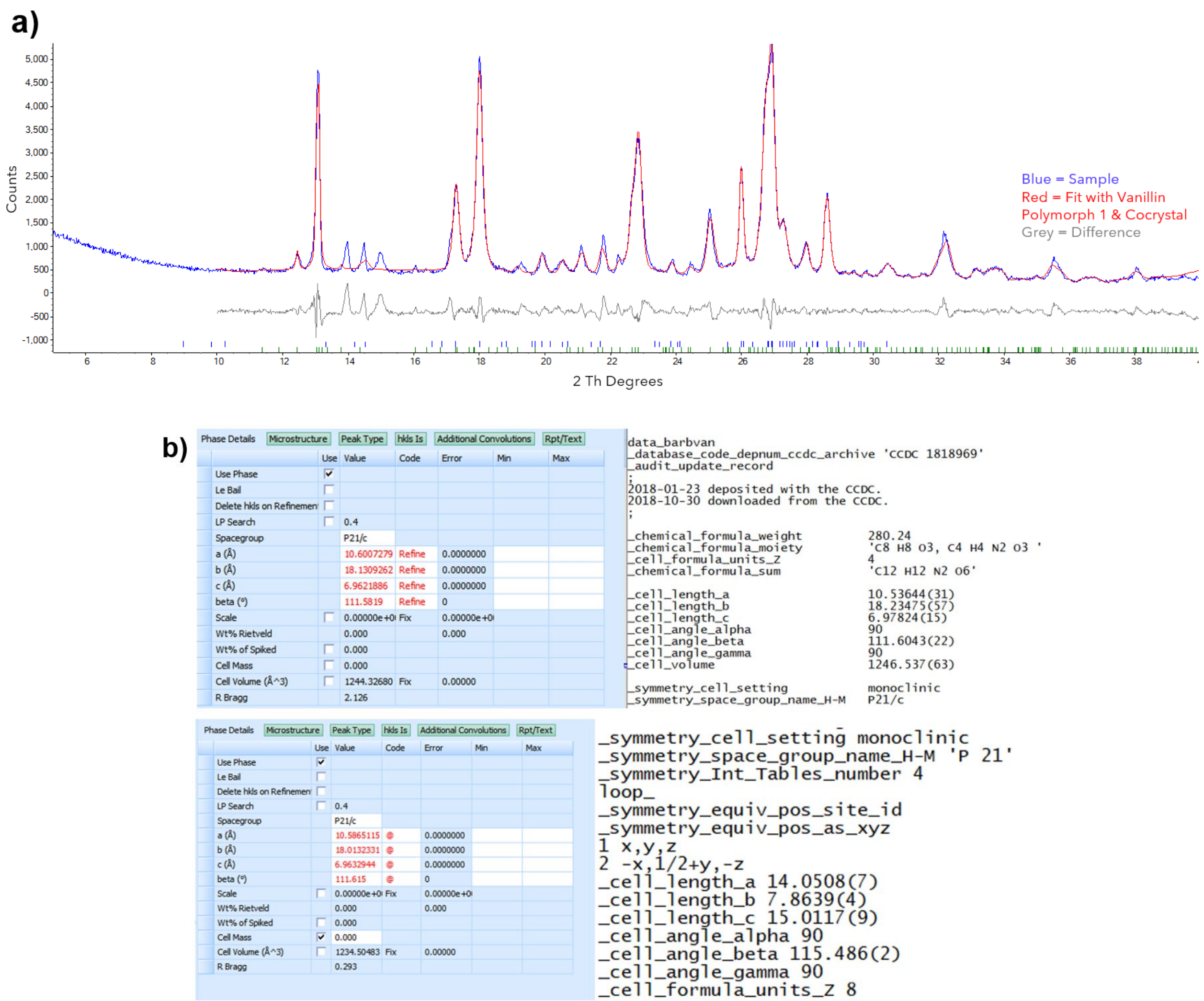

Figure S23. a) PXRD pattern and fitting of yellow compound formed from pre-milled wet vanillin and barbituric acid pressed together, b) additional PXRD fit analysis data.

PXRD shows a heterogeneous mixture of cocrystal and vanillin (polymorph 1) and a small amount of an unidentified crystalline compound (see 14 deg 2theta, Figure S22a). We did not observe vanillin polymorph 2. We did not explore further as data was sufficient to confirm that yellow coloration corresponded to co-crystal formation.

9. REGRESSION ANALYSIS FOR KINETIC FITS

Initial condition was set for the consumption of reactants N_1, and the formation of product N_2. The function outputs an array containing product concentration N_2 at each time point which is used in the regression analysis to fit the model to the experimental data. To estimate the kinetic parameters, a script implemented in Matlab uses a loop to perform the fitting for each of the ten experiments individually. A fit function in Matlab was used to perform nonlinear regression, fitting the model to the experimental yield data $Y(t)$. An initial linear fit (ploy1) was performed on the first two data points to estimate the initial rate and provide insights into the early-stage kinetics. The fit function was used such that k_A is fixed for a first-order reaction with initial guess of $k_B =$

$0.5\,x\,10^{-6}\,s^{-1}$. The objective of the regression analysis is to minimize the sum of squared residuals between the experimental measurements and model predictions while satisfying physical constraints. The objective function $\mathcal{F}$ for the nonlinear least squared is defined as;

$$\mathcal{F}(\theta) = \sum_{i=1}^{M}\sum_{j=1}^{N_i} w_{i,j}\left[y_{i,j}^{exp} - y_{i,j}^{model}(\theta)\right]^2 \quad (0)$$

where $\theta = [k_A, k_B, n]$ is the parameter vector, M is the number of experiments, N_i is the number of measurements in experiments i. $w_{i,j}$ are the weighting factors, $y_{i,j}^{exp}$ are the experimental measurement and $y_{i,j}^{model}(\theta)$ are the model predictions. Parameter optimization bounds used to ensure physically meaningful values and convergence was given as;

$$6\,x\,10^{-7} \le k_B \le 6\,x\,10^{-5}\,s^{-1}$$

This optimization employs a trust-region algorithm that creates a region around the current parameter where we "trust" the quadratic approximation, solves the coupled ordinary differential equations, compute the objective function, and update parameters via the trust-region step until convergence criteria are met. The explicit Euler method which allows for flexible time discretization was utilized to numerically solve the ODEs that govern the reaction kinetics described in the article. Fitted parameters k_B and n were extracted, and the confidence interval was set at 50% to assess the precision of the estimates. The model was evaluated with the fitted parameters over a time grid to generate continuous predicted yield curves. Pseudocode used for this analysis is shown in Figure S24 and resulting R^2 values are given in Table S1.

Algorithm 1 Nonlinear Regression

Require:
Experimental data: $\{t_j, y_j^{\text{exp}}\}_{j=1}^{M}$
Initial conditions: $N_0 = 100$
Parameter bounds: $[k_A^{\text{min}}, k_A^{\text{max}}]$, $[k_B^{\text{min}}, k_B^{\text{max}}]$, , $[n^{\text{min}}, n^{\text{max}}]$
Initial parameter guesses: $\boldsymbol{\theta}_0 = [k_{A,0}, k_{B,0}, n_0]^T$
Ensure:
Optimal parameters: $\boldsymbol{\theta}^* = [k_A^*, k_B^*, n^*]^T$
Model predictions: $\{y_j^{\text{model}}\}_{j=1}^{M}$
Statistical metrics: R^2, RMSE, confidence intervals
procedure Analysis
  Data Preprocessing:
  for each experiment i **do**
    Convert time to seconds: $t_i \leftarrow t_i \times 60$
    if frequency = 30 Hz **then**
      Scale time: $t_i \leftarrow t_i \times \frac{25}{30}$
    end if
  end for
  Initialize trust-region optimizer with $\boldsymbol{\theta}_0$ (creates region around current parameter where we "trust" the quadratic approximation)
  while not converged **do**
    Solve coupled ODEs
    Compute objective function $\mathcal{J}(\boldsymbol{\theta})$
    Update parameters via trust-region step
    Check convergence criteria
  end while
  Statistical Analysis:
  Compute Jacobian and covariance matrix
  Compute confidence intervals
  Calculate R^2 and RMSE
  Initial Rate Analysis:
  Perform linear regression on initial points to calculate the initial rate
end procedure

Figure S24. Pseudocode for regression analysis.

Table S1. R^2 values for kinetic fits from regression analysis.

Milling Media Material	Ball Size (mm)	Frequency (Hz)	Regression R^2 Value
SS	15	25	0.9315
SS	12.7	25	0.6258
ZR	12.7	25	0.9986
AL	12.7	25	0.9836
PTFE	12.7	25	0.9223
SS	15	30	0.8636
SS	12.7	30	0.9862
ZR	12.7	30	0.9714
AL	12.7	30	0.9299
PTFE	12.7	30	0.8191

10. TEMPERATURE MEASUREMENTS

In a previous report by Hutchings and co-workers, it was noted that the milling ball experienced the greatest temperature change during milling due to the formation of the cohesive state coating the ball that occurs during the feedback period.[3] As such, they attributed the observed increase in reaction rate during this period to the increase in temperature. While the appearance of the "snowball" was inconsistent in our experiments, we performed secondary experiments measuring the temperature of the jar and ball at different time points over a period of 120 min to see if we observe the same elevated temperatures across the different systems. The temperature measurements for the milling ball in different systems are shown in Figures S25-30.

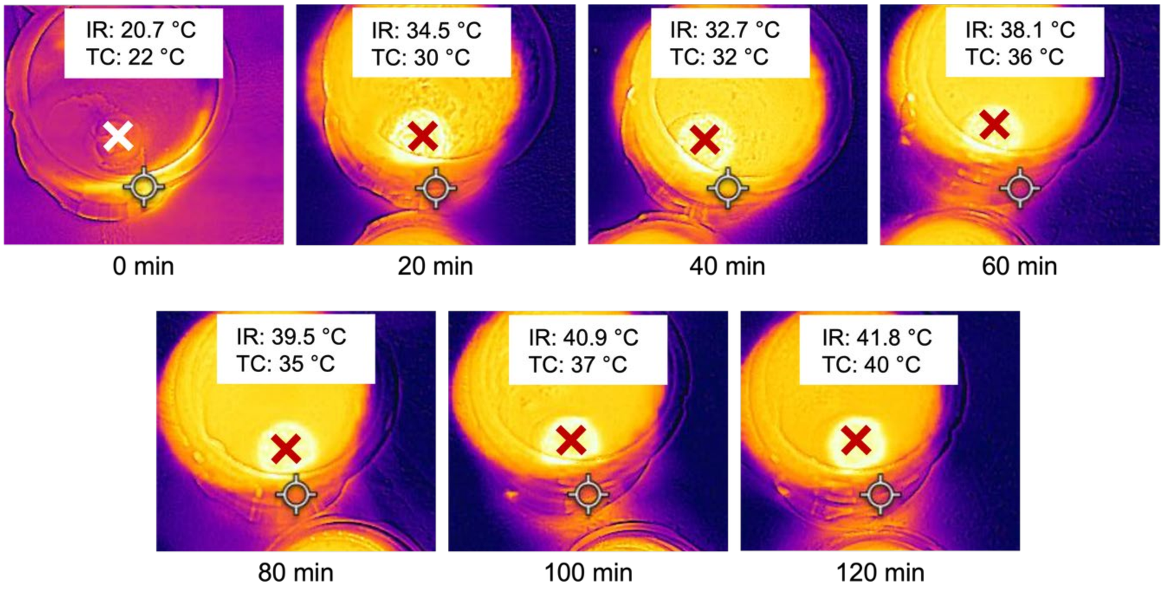

Figure S25. Thermal images of SS ball (12.7 mm) used in 25 mL SS jar at different points of milling at 30 Hz. The thermal camera (IR) and thermocouple (TC) temperature readings are given. The location of the laser spot is indicated by an X mark.

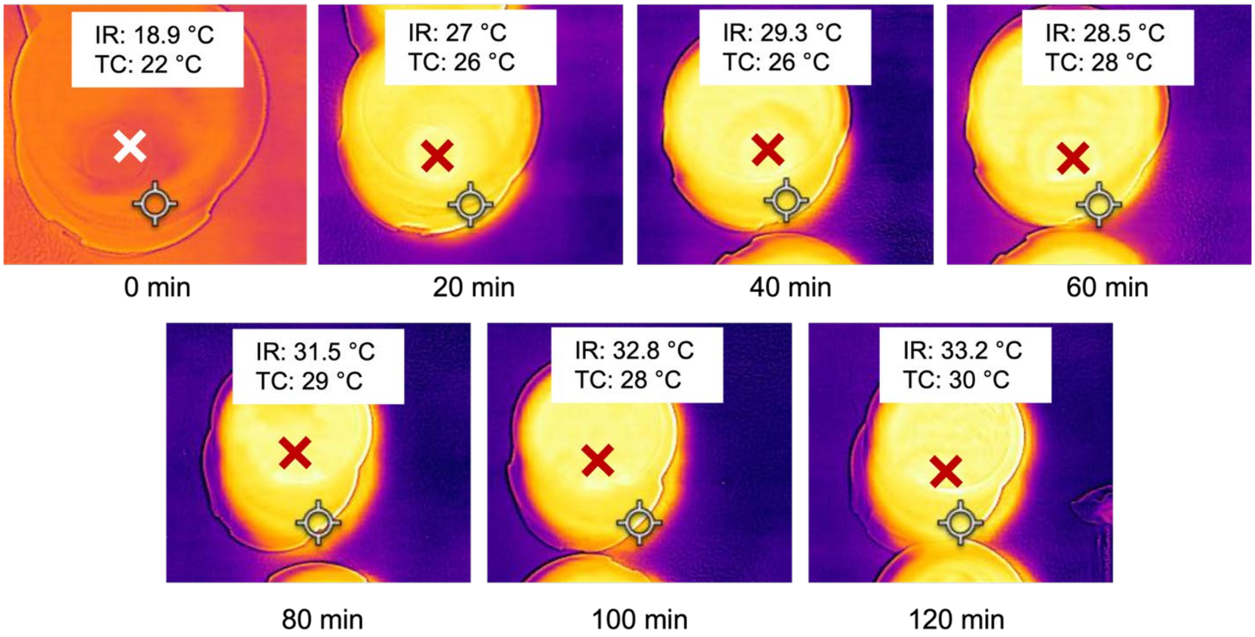

Figure S26. Thermal images of PTFE ball (12.7 mm) in a 25 mL PTFE jar at different points of milling at 30 Hz. The thermal camera (IR) and thermocouple (TC) temperature readings are given. The location of the laser spot is indicated by an X mark.

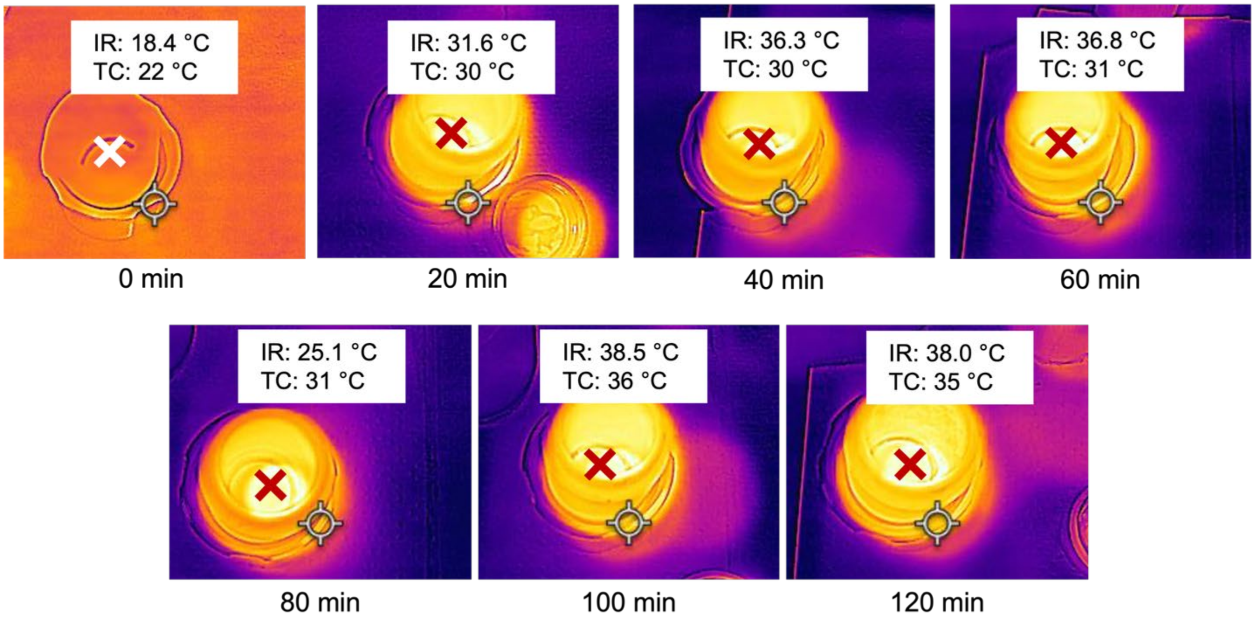

Figure S27. Thermal images of PTFE ball (16 mm) in jar insert with PTFE body and SS ends at different points of milling at 30 Hz. The thermal camera (IR) and thermocouple (TC) temperature readings are given. The location of the laser spot is indicated by an X mark.

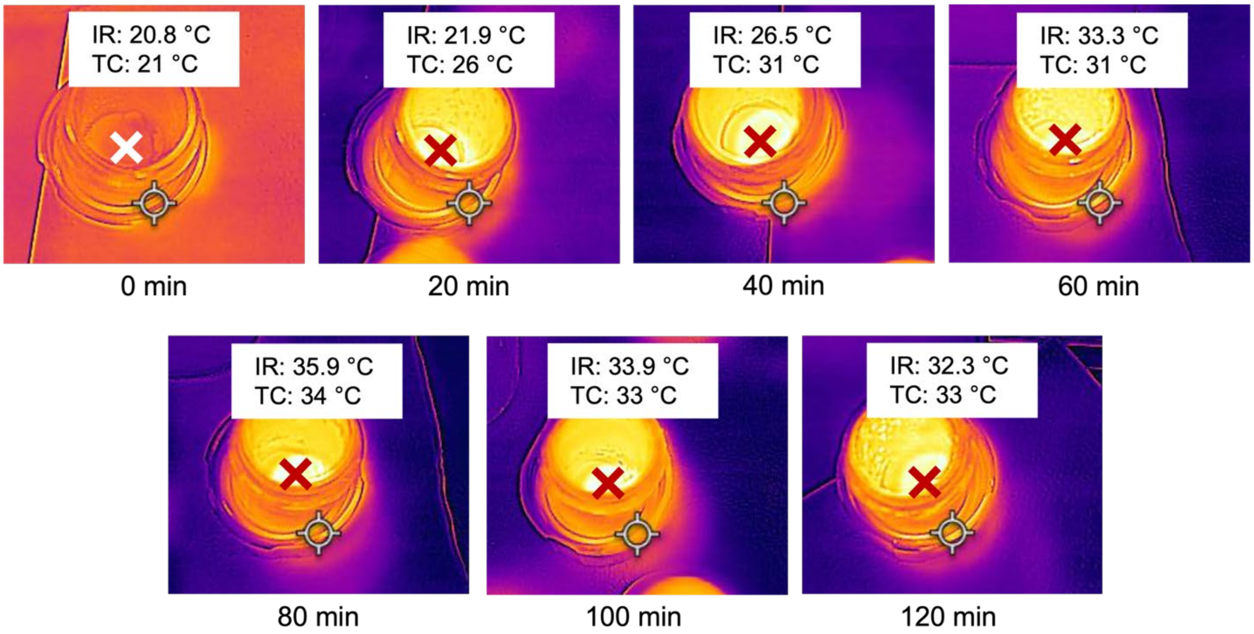

Figure S28. Thermal images of PTFE ball (16 mm) in jar insert with SS body and PTFE ends at different points of milling at 30 Hz. The thermal camera (IR) and thermocouple (TC) temperature readings are given. The location of the laser spot is indicated by an X mark.

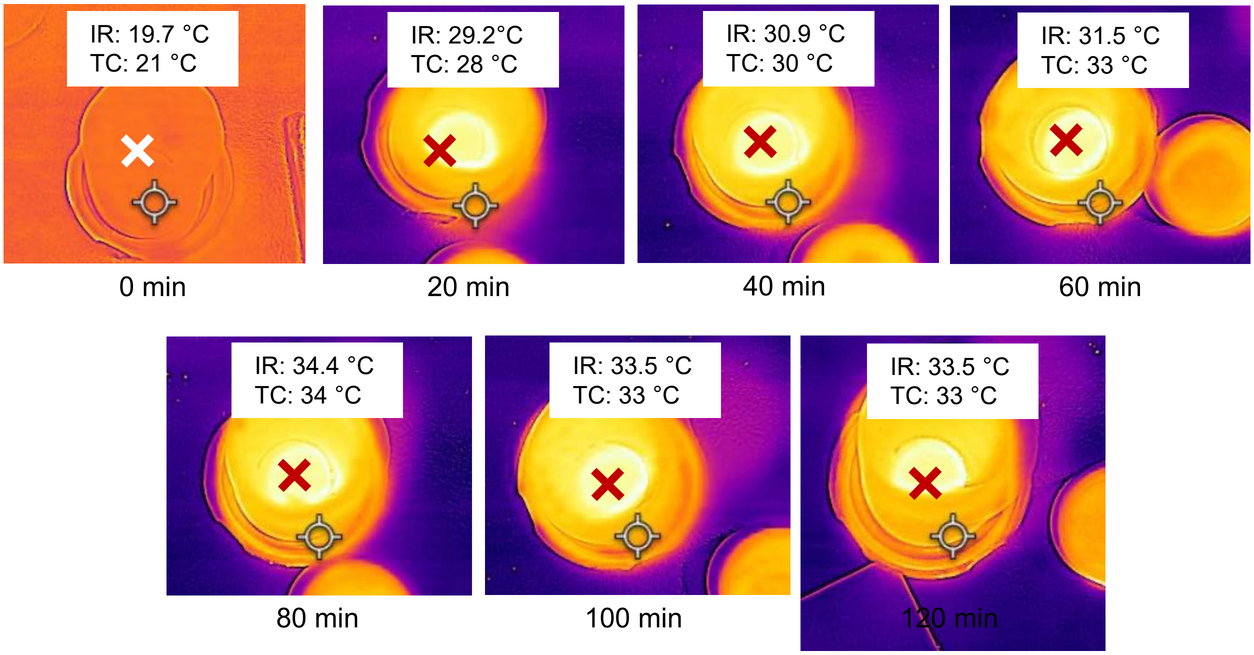

Figure S29. Thermal images of PTFE ball (16 mm) in PTFE jar insert with at different points of milling at 30 Hz. The thermal camera (IR) and thermocouple (TC) temperature readings are given. The location of the laser spot is indicated by an X mark.

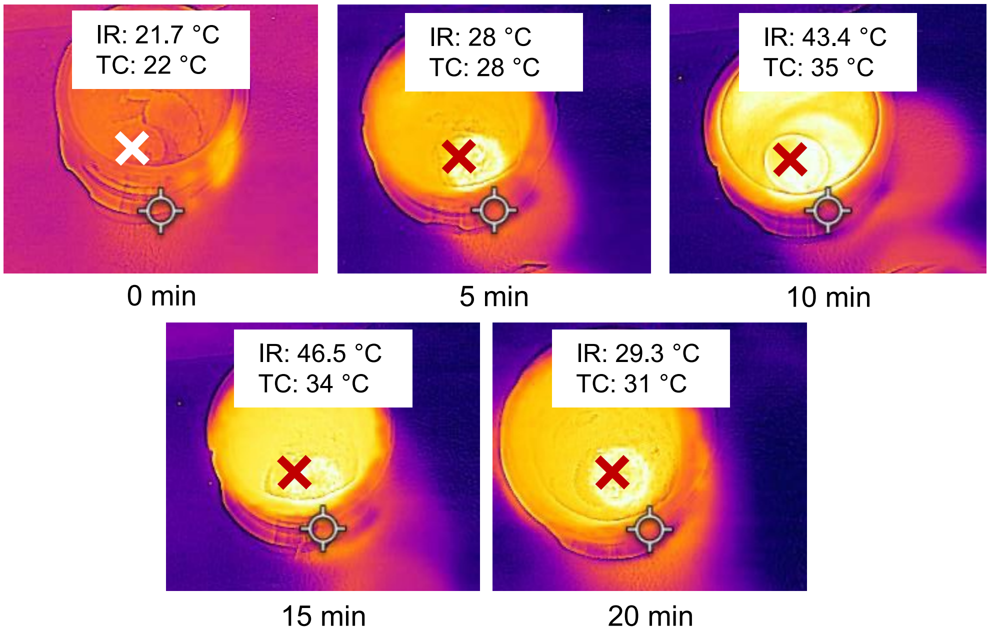

Figure S30. Thermal images of SS ball (12.7 mm) in a 25 mL SS jar at different points of milling at 30 Hz in autocatalysis experiments. The thermal camera (IR) and thermocouple (TC) temperature readings are given. The location of the laser spot is indicated by an X mark.

11. AUTOCATALYSIS EXPERIMENTS

The autocatalytic nature of the Knoevenagel condensation reaction in ball milling conditions have been reported in previous literature.[4,5] This effect was attributed to the product initiating further product crystallization in the solid-state reaction. [5] To prove that the kinetics of the reaction is influenced by the presence of the product, we compare the kinetic profiles of the reaction at the same conditions with and without added product (Figure S34). Results show that the induction period is shorter for the reaction in the presence of the product.

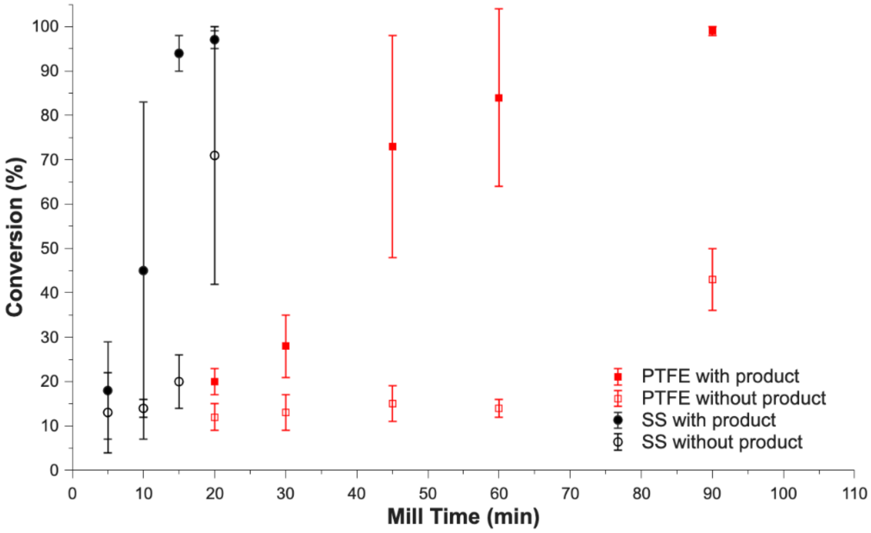

Figure S33. Reaction kinetics with and without added product using 25 mL PTFE jar and 12.7 mm PTFE ball (square) and 25 mL SS jar and 12.7 mm ball (circle) at 30 Hz. Each data point is an average of three measurements and error is given as standard deviation.

12. DOI LINK TO RAW DATA FILES

Raw data files for results presented in the manuscript and herein are available at the following DOI:10.6084/m9.figshare.28038410

13. REFERENCES

(1) Floyd, K.; Gonnet, L.; Friščić, T.; Batteas, J. The role of the milling environment on the copper-catalysed mechanochemical synthesis of Tolbutamide. *RSC Mechanochemistry* **2024**, *1* (3), 289-295, 10.1039/D4MR00031E. DOI: 10.1039/D4MR00031E.

(2) Floyd, K.; Batteas, J. D.; Nwoye, E.; Felts, J. R.; Gonzalez, P.; Mella, L. An Exchangeable Mulitcomponent Ball-Milling Reactor System (aka. EMBRS). US Provisional Patent Application #63/749,494, January 25, 2025.

(3) Hutchings, B. P.; Crawford, D. E.; Gao, L.; Hu, P.; James, S. L. Feedback Kinetics in Mechanochemistry: The Importance of Cohesive States. *Angew. Chem. Int. Ed.* **2017**, *56* (48), 15252-15256.

(4) Trotzki, R.; Hoffmann, M. M.; Ondruschka, B. Studies on the solvent-free and waste-free Knoevenagel condensation. *Green Chem.* **2008**, *10* (7), 767-772, 10.1039/B801661E. Kaupp, G.; Reza Naimi-Jamal, M.; Schmeyers, J. Solvent-free Knoevenagel condensations and Michael additions in the solid state and in the melt with quantitative yield. *Tetrahedron* **2003**, *59* (21), 3753-3760.

(5) Burmeister, C. F.; Schmidt, R.; Jacob, K.; Breitung-Faes, S.; Stolle, A.; Kwade, A. Effect of stressing conditions on mechanochemical Knoevenagel synthesis. *Chemical Engineering Journal* **2020**, *396*.
